# Results of next‐generation sequencing gene panel diagnostics including copy‐number variation analysis in 810 patients suspected of heritable thoracic aortic disorders

**DOI:** 10.1002/humu.23565

**Published:** 2018-07-12

**Authors:** Eline Overwater, Luisa Marsili, Marieke J.H. Baars, Annette F. Baas, Irma van de Beek, Eelco Dulfer, Johanna M. van Hagen, Yvonne Hilhorst‐Hofstee, Marlies Kempers, Ingrid P. Krapels, Leonie A. Menke, Judith M.A. Verhagen, Kak K. Yeung, Petra J.G. Zwijnenburg, Maarten Groenink, Peter van Rijn, Marjan M. Weiss, Els Voorhoeve, J. Peter van Tintelen, Arjan C. Houweling, Alessandra Maugeri

**Affiliations:** ^1^ Department of Clinical Genetics VU University Medical Center Amsterdam the Netherlands; ^2^ Department of Clinical Genetics, Academic Medical Center University of Amsterdam Amsterdam the Netherlands; ^3^ Medical Genetics Unit Tor Vergata University Hospital Rome Italy; ^4^ Department of Medical Genetics University Medical Center Utrecht Utrecht the Netherlands; ^5^ Department of Genetics University Medical Center Groningen Groningen the Netherlands; ^6^ Department of Clinical Genetics Leiden University Medical Center Leiden the Netherlands; ^7^ Department of Human Genetics Radboud University Medical Center Nijmegen the Netherlands; ^8^ Department of Clinical Genetics Maastricht University Medical Center Maastricht the Netherlands; ^9^ Department of Pediatrics, Academic Medical Center University of Amsterdam Amsterdam the Netherlands; ^10^ Department of Clinical Genetics Erasmus University Medical Center Rotterdam the Netherlands; ^11^ Department of Surgery, Institute for Cardiovascular Research VU University Medical Center Amsterdam the Netherlands; ^12^ Department of Physiology, Institute for Cardiovascular Research VU University Medical Center Amsterdam the Netherlands; ^13^ Department of Cardiology and Radiology, Academic Medical Center Amsterdam University of Amsterdam Amsterdam the Netherlands

**Keywords:** copy‐number variations, eXome hidden Markov model, genetics, thoracic aortic aneurysm, thoracic aortic dissection

## Abstract

Simultaneous analysis of multiple genes using next‐generation sequencing (NGS) technology has become widely available. Copy‐number variations (CNVs) in disease‐associated genes have emerged as a cause for several hereditary disorders. CNVs are, however, not routinely detected using NGS analysis. The aim of this study was to assess the diagnostic yield and the prevalence of CNVs using our panel of Hereditary Thoracic Aortic Disease (H‐TAD)‐associated genes. Eight hundred ten patients suspected of H‐TAD were analyzed by targeted NGS analysis of 21 H‐TAD associated genes. In addition, the eXome hidden Markov model (XHMM; an algorithm to identify CNVs in targeted NGS data) was used to detect CNVs in these genes. A pathogenic or likely pathogenic variant was found in 66 of 810 patients (8.1%). Of these 66 pathogenic or likely pathogenic variants, six (9.1%) were CNVs not detectable by routine NGS analysis. These CNVs were four intragenic (multi‐)exon deletions in *MYLK*, *TGFB2*, *SMAD3*, and *PRKG1*, respectively. In addition, a large duplication including *NOTCH1* and a large deletion encompassing *SCARF2* were detected. As confirmed by additional analyses, both CNVs indicated larger chromosomal abnormalities, which could explain the phenotype in both patients. Given the clinical relevance of the identification of a genetic cause, CNV analysis using a method such as XHMM should be incorporated into the clinical diagnostic care for H‐TAD patients.

## 1 BACKGROUND

Over the last decade, advances in clinical genetics have led to the identification of disease‐associated genes at a rapid pace. Especially when surveillance, early detection, and/or treatment provide health benefits for the index patient and at‐risk relatives, identification of an underlying genetic cause is highly relevant. Therefore, recommendations for genetic counseling and DNA testing are increasingly being incorporated into clinical guidelines (Ackerman et al., 2011; Eccles et al., 2016). Thoracic aortic aneurysms and aortic dissections (TAAD) are a significant cause of sudden death at young age and is an example of a disease where screening of at‐risk relatives can be lifesaving (Hoyert, Arias, Smith, Murphy, & Kochanek, 2001; Olsson, Thelin, Stahle, Ekbom, & Granath, 2006). Because aortic aneurysms are often asymptomatic and aortic dissections are often fatal and preventable by timely surgical intervention, the identification and clinical screening of at‐risk relatives are clinically highly relevant and recommended (Hiratzka et al., 2010). In the majority of cases, TAAD is a sporadic occurrence, associated with, among others, hypertension, bicuspid aortic valve, and older age. However, in approximately 20% of cases TAAD is reported to be familial (FTAAD), often with an autosomal dominant pattern of inheritance with incomplete penetrance (Biddinger, Rocklin, Coselli, & Milewicz, 1997; Coady et al., 1999; Robertson et al., 2016). TAAD that is caused by a pathogenic variant in one of the disease‐associated genes (Hereditary Thoracic Aortic Disease (H‐TAD)) can be subdivided in nonsyndromic and syndromic aortic disease. The phenotypic manifestations of both syndromic and nonsyndromic H‐TAD are highly variable, both within and between families. Syndromic H‐TAD is only diagnosed in a minority of cases and includes, among others, Marfan syndrome (MIM# 154700), Loeys–Dietz syndrome (MIM# 609192, MIM# 610168, MIM# 613795, MIM# 614816, and MIM# 615582), and vascular Ehlers–Danlos syndrome (MIM# 130050). The genes most frequently associated with nonsyndromic H‐TAD are involved in smooth‐muscle cell function (*ACTA2*, MIM# 611788, *MYH11*, MIM# 132900, and *MYLK*, MIM# 613780). Of note, variants in genes originally associated with syndromic H‐TAD have also been reported in patients presenting with apparently nonsyndromic H‐TAD (Gago‐Diaz et al., 2014; Regalado et al., 2011, 2016). Given the incomplete penetrance and the highly variable age of onset within both heritable and sporadic TAAD (Campens et al., 2015; Coady et al., 1999; Khalique et al., 2009; Robertson et al., 2016), follow‐up of at‐risk relatives with normal aortic diameters at initial cardiologic screening is important. The identification of a pathogenic variant in a TAAD patient allows for targeted screening of relatives and enables prenatal and preimplantation genetic diagnosis. In addition, specific recommendations on imaging, surgical, and pharmacological treatment based on the underlying genetic cause are emerging (den Hartog et al., 2016; Franken et al., 2015; D. Milewicz et al., 2016). A causative variant can be identified in approximately 20% of FTAAD families (D. M. Milewicz, Regalado, Shendure, Nickerson, & Guo, 2014). Next‐generation sequencing (NGS) allows for the rapid analysis of multiple genes in a diagnostic setting at relatively low costs. Therefore, DNA testing is increasingly offered to TAAD patients. The majority of the detected variants are single‐nucleotide changes. CNVs have emerged as a relevant cause for several genetic disorders including cancer, intellectual disability, and neuropsychiatric disorders (Pollack et al., 2002; Shlien & Malkin, 2010; Thapar & Cooper, 2013). Routine diagnostic variant‐calling analysis by (short reads‐)NGS technology is not suitable for detecting CNVs. Therefore, CNVs may be missed unless additional testing is performed, for example, by multiplex ligation‐dependent probe amplification (MLPA) or targeted array analysis. However, these tests are often not routinely performed and/or do not include all the relevant genes. The detection of CNVs in NGS sequencing data using statistical and computational tools is an alternative approach. The eXome hidden Markov model (XHMM) is one of several algorithms developed for the detection of CNVs through NGS data (Fromer & Purcell, 2014; Fromer et al., 2012). XHMM has identified (potential) causative CNVs in, for example, patients with Parkinson's disease, autism spectrum disorders, and rare diseases like Joubert syndrome and very early onset inflammatory bowel disease (Kelsen et al., 2015; Koyama et al., 2017; Poultney et al., 2013; Spataro et al., 2017). The aim of this study was to assess both the diagnostic yield of our panel of H‐TAD‐associated genes and the prevalence of CNVs in these genes. Here, we present the results of routine NGS analysis (variant‐calling analysis) and XHMM analysis on the NGS sequencing data of the largest series of TAAD patients described so far (*n* = 810) referred for analyses of the H‐TAD panel. In addition, we provide an overview of the clinical data of patients with a pathogenic or likely pathogenic variant, with a special focus on patients with CNVs. The results of this study underline the importance of CNV analysis in routine diagnostic testing in patients with H‐TAD.

## 2 METHODS

### 2.1 Genetic data

DNA diagnostics was performed at the Department of Clinical Genetics at the VU University Medical Center (VUmc, Amsterdam, the Netherlands) from March 2015 to June 2017. The routine NGS panel included *ACTA2, COL3A1, EFEMP2, ELN, FBN1, FBN2, MYH11, MYLK, NOTCH1, PLOD1, PRKG1, SCARF2, SKI, SLC2A10, SMAD2, SMAD3, SMAD4, TGFB2, TGFB3, TGFBR1*, and *TGFBR2*. Since October 2016, the *BGN* gene was added to the panel (analyzed in 166 patients), while *SCARF2*, which was not associated with TAD but had previously been selected in view of a possible differential diagnosis ‘Congenital contractural arachnodactyly’ and ‘Van den Ende–Gupta syndrome,’ was excluded from routine analysis. The previously described bioinformatics read‐depth‐based tool XHMM was used for CNV detection in the NGS sequencing data. CNV confirmation was performed using either a home‐made MLPA test, in combination with the P300 or the P200 MLPA kit of MRC Holland, or an SNP array. Detailed information on the analyzed genes and applied methodologies are available in the Supporting Materials and Methods.

### 2.2 Clinical data

Informed consent for NGS gene panel analysis was obtained from all 810 patients after genetic counseling by the referring physician. The main reasons for analysis of this gene panel include familial or early onset aortic aneurysms or dissections or signs of generalized connective tissue disorders. The majority of patients was referred by a clinical geneticist who frequently participated in a multidisciplinary team specialized in connective tissue disorders. A standardized survey was sent to the referring physicians in order to collect the medical data of patients carrying an identified genetic variant (including ophthalmologic and cardiologic findings, family history, and physical examination). Written informed consent was obtained from the patients and/or their parents with an aberration detected by XHMM, as more detailed medical data were published. Under Dutch law, assessment of the study protocol by our ethics committee was not indicated because only genetic and clinical data collected during regular patient care were used.

## 3 RESULTS

A pathogenic or likely pathogenic variant in an H‐TAD‐associated gene was identified in 66 of 810 index patients (8.1%). Of these, 60 (90.9%) were identified using routine NGS panel analysis (variant‐calling analysis). In the other six cases (9.1%), a pathogenic or likely pathogenic CNV was detected using XHMM. In 84 patients (10.4%), only variants of unknown significance (VUS) were identified. No pathogenic or likely pathogenic variants and/or VUS were identified in 660 patients (81.5%). The mean age at DNA diagnostics of index patients with a pathogenic or likely pathogenic variant was 35 years (median 36, range 0–77). The mean age of the remaining patients was 46 years (median 49, range 0–78). There was a male preponderance in index patients with a pathogenic or likely pathogenic variant, VUS, or without a VUS or pathogenic variant (68%, 64%, and 67%, respectively).

### 3.1 Genetic and clinical data in patients with variants identified by variant‐calling analysis

Table 1 provides an overview of the molecular data of the 60 pathogenic or likely pathogenic variants identified by variant‐calling analysis. Of these variants, 37 (62%) have not been described previously and all of them were unique. Heterozygous pathogenic or likely pathogenic variants were identified in *FBN1* (*N* = 18, 30%), *ACTA2* (*N* = 8, 13.3%), *SMAD3* (*N* = 7, 11.7%), *COL3A1* (*N* = 6, 10%), *TGFB2* (*N* = 4, 6.7%), *TGFBR1* (*N* = 3, 5%), *TGFBR2* (*N* = 3, 5%), *FBN2* (*N* = 3, 5%), *MYH11* (*N* = 2, 3.3%), *TGFB3* (*N* = 2, 3.3%), *PRKG1* (*N* = 1, 1.7%), and *NOTCH1* (*N* = 1, 1.7%). Homozygous pathogenic *SLC2A10* variants were identified in two patients (3.3%). No (likely) pathogenic variants were found in *BGN*, *EFEMP2*, *ELN*, *PLOD1*, *SKI*, *SMAD2*, and *SMAD4*. In addition, 90 VUS were identified (patients 9, 52, 67–150; Table 1 and Supporting Information Table S1). In six patients (patients 9 and 52 in Table 1 and Supporting Information Table S1; and patients 69, 75, 90, and 127 in Supporting Information Table S1), two VUS (in different genes) were identified. An overview of the clinical data of all 60 patients with a pathogenic or likely pathogenic variant identified by variant‐calling analysis is provided in Table 2. The clinical data of patients 67–150 with a VUS are available in Supporting Information Table S2.

**Table 1:** Summary of the genetic features of patients with a pathogenic or likely pathogenic variant detected by variant‐calling analysis of 21 H‐TAD genes

Patient	Gene	Nucleotide change	Protein change	Effect	Domain	Conservation	SIFT/MutationTaster/Polyphen‐2/Grantham distance	MAF ExAC	Segregation analysis^a^	Reference
1	***ACTA2***	**c.115C > T**	**p.(Arg39Cys)**	**Missense**	**Actin**	**Baker's yeast** b	**+ / + / − / 180**	**Absent**	**yes**	**(** **Hoffjan et al.,** 2011 **)**
2	***ACTA2***	**c.116G > A**	**p.(Arg39His)**	**Missense**	**Actin**	**Baker's yeast** b	**+ / + / − / 29**	**Absent**	**yes**	**(** **Guo et al.,** 2009 **)**
3	*ACTA2*	c.179C > A	p.(Ala60Glu)	Missense	Actin	Baker's yeast^b^	+ / + / + / 107	Absent	n.a.	Novel
4	*ACTA2*	c.419C > T	p.(Ala140Val)	Missense	Actin	Baker's yeast^b^	+ / + / ±/ 64	Absent	yes	(Lerner‐Ellis et al., 2014)
5^c^	***ACTA2***	**c.445C > T**	**p.(Arg149Cys)**	**Missense**	**Actin**	**Baker's yeast** b	**+ / + / + / 180**	**Absent**	**yes**	**(** **Guo et al.,** 2007 **)**
6	*ACTA2*	c.835A > G	p.(Thr279Ala)	Missense	Actin	Baker's yeast^b^	+ / + / **−** / 58	Absent	n.a.	Novel
7	*ACTA2*	c.854T > C	p.(Met285Thr)	Missense	Actin	Baker's yeast^b^	+ / + / ± / 81	Absent	n.a.	Novel
8	*ACTA2*	c.1120C > T	p.(Arg374Cys)	Missense	Actin	*C. elegans* (FCUT Baker's yeast)	+ / + / **−** / 180	1 / 121346	n.a.	Novel
9^d^	***COL3A1***	**c.318_325del**	p.(Pro107Argfs*13)	**Frameshift (NMD expected)**	**NA**	**NA**	**NA**	**Absent**	**n.a**.	**Novel**
10	***COL3A1***	**c.555del**	p.(Gly186Valfs*36)	**Frameshift (NMD expected)**	**NA**	**NA**	**NA**	**Absent**	**yes, incomplete penetrance**	**(** **Pepin et al.,** 2014 **;** **Schwarze et al.,** 2001 **)**
11	***COL3A1***	**c.971G > A**	**p.(Gly324Asp)**	**Missense**	**Triple helix**	**Chicken** b	**+ / + / ± / 94**	**Absent**	***de novo*** e	**Novel**
12	***COL3A1***	**c.2050G > A**	**p.(Gly684Arg)**	**Missense**	**Triple helix**	**Chicken** b	**+ / + / + / 125**	**Absent**	**yes**	**Novel**
13	***COL3A1***	**c.3219_3222dup**	p.(Ala1075Trpfs*20)	**Frameshift (NMD expected)**	**NA**	**NA**	**NA**	**Absent**	**Maternally inherited**	**Novel**
14	***COL3A1***	**c.3446G > A**	**p.(Gly1149Asp)**	**Missense**	**Triple helix**	**Chicken** b	**+ / + / + / 94**	**Absent**	**n.a**.	**(** **Frank et al.,** 2015 **)**
15	*FBN1*	c.32T > G	p.(Leu11Arg)	Missense	Signal peptide	Dog^b^	+ / + / + / 102	Absent	n.a.	(Baetens et al., 2011)
16	***FBN1***	**c.439C > T**	p.(Gln147*)	**Nonsense (NMD expected)**	**NA**	**NA**	**NA**	**Absent**	**n.a**.	**Novel**
17	***FBN1***	**c.986dup**	p.(Asp330Argfs*18)	**Frameshift (NMD expected)**	**NA**	**NA**	**NA**	**Absent**	**n.a**.	**Novel**
18	***FBN1***	**c.2177A > G**	**p.(Glu726Gly)**	**Missense**	**EGF‐like 11**	**Tetraodon** b	**+ / + / + / 98**	**Absent**	**n.a**.	**(** **Stheneur et al.,** 2009 **)**
19	*FBN1*	c.2645C > T	p.(Ala882Val)	Missense	TB 4	Tetraodon^b^	+ / + / + / 64	Absent	n.a.	(Aragon‐Martin et al., 2010; Comeglio et al., 2007; Howarth, Yearwood, & Harvey, 2007; Hung et al., 2009; B. Loeys et al., 2004; Robinson et al., 2012)
20	***FBN1***	**c.2660G > A**	**p.(Cys887Tyr)**	**Missense**	**TB 4**	**Tetraodon** b	**+ / + / + / 194**	**Absent**	**n.a**.	**Novel**
21	***FBN1***	**c.2668T > C**	**p.(Cys890Arg)**	**Missense**	**TB 4**	**Tetraodon** b	**+ / + / + / 180**	**Absent**	**n.a**.	**(** **Collod‐Beroud et al.,** 2003 **;** **Kielty, Rantamaki, Child, Shuttleworth, & Peltonen,** 1995 **)**
22	***FBN1***	**c.2953G > A**	**p.(Gly985Arg)**	**Missense**	**TB 5**	**Tetraodon** b	**+ / + / + / 125**	**Absent**	**n.a**.	**(** **Faivre et al.,** 2009 **;** **Howarth et al.,** 2007 **; B**. **Loeys, Nuytinck, Delvaux, De Bie, & De Paepe,** 2001 **;** **Rommel et al.,** 2005 **;** **Turner et al.,** 2009 **;** **Yoo et al.,** 2010 **)**
23	***FBN1***	**c.3152T > G**	**p.(Phe1051Cys)**	**Missense**	**EGF‐like 15**	**Tetraodon** b	**+ / + / + / 205**	**Absent**	**n.a**.	**Novel**
24	***FBN1***	**c.3373C > T**	p.(Arg1125*)	**Nonsense (NMD expected)**	**NA**	**NA**	**NA**	**Absent**	**yes**	**(** **Attanasio et al.,** 2008 **;** **Comeglio et al.,** 2007 **;** **Hung et al.,** 2009 **;** **Magyar et al.,** 2009 **;** **Rommel et al.,** 2005 **;** **Sheikhzadeh et al.,** 2012 **;** **Stheneur et al.,** 2009 **)**
25	***FBN1***	**c.4987T > C**	**p.(Cys1663Arg)**	**Missense**	**EGF‐like 28**	**Zebrafish** b	**+ / + / + / 180**	**Absent**	**n.a**.	**(** **Dietz, Saraiva, Pyeritz, Cutting, & Francomano,** 1992 **;** **Stheneur et al.,** 2009 **;** **Yoo et al.,** 2010 **)**
26	***FBN1***	**c.5015del**	p.(Cys1672Leufs*10)	**Frameshift (NMD expected)**	**NA**	**NA**	**NA**	**Absent**	**n.a**.	**Novel**
27	***FBN1***	**c.5699G > C**	**p.(Cys1900Ser)**	**Missense**	**EGF‐like 32**	**Zebrafish** b	**+ / + / + / 112**	**Absent**	**n.a**.	**(** **Stheneur et al.,** 2009 **)**
28	***FBN1***	**c.6031T > C**	**p.(Cys2011Arg)**	**Missense**	**EGF‐like 34**	**Zebrafish** b	**+ / + / + / 180**	**Absent**	***de novo*** e	**Novel**
29	***FBN1***	**c.6942C > G**	p.(Tyr2314*)	**Nonsense (NMD expected)**	**NA**	**NA**	**NA**	**Absent**	***de novo*** e	**Novel**
30	*FBN1*	c.7708G > A	p.(Glu2570Lys)	Missense	EGF‐like 45	Tetraodon^b^	+ / + / + / 56	Absent	n.a.	(Arbustini et al., 2005; Attanasio et al., 2008; Soylen et al., 2009)
31	*FBN1*	c.8188C > T	p.(Arg2730Trp)	Missense	C‐terminal domain	Tetraodon^b^	+ / + / + / 101	Absent	n.a.	Novel
32	***FBN1***	**c.8578_8579dup**	p.(Asp2860Glufs*4)	**Frameshift (NMD not expected)**	**Asprosin chain**	**NA**	**NA**	**Absent**	**n.a**.	**Novel**
33	*FBN2*	c.3812G > C	p.(Gly1271Ala)	Missense	EGF‐like 19	Chicken^b^	+ / + / + / 60	Absent	n.a.	(Buchan et al., 2014)
34	*FBN2*	c.3889G > A	p.(Gly1297Ser)	Missense	EGF‐like 20	Chicken^b^	+ / + / + / 56	2 / 121372	Paternally inherited	Novel
35	*FBN2*	c.7526_7527del	p.0	Frameshift (NMD confirmed)	NA	NA	NA	Absent	n.a.	Novel
36	*MYH11*	c.3315‐5G > A	p.?	Splice (NMD not expected)	Coiled coil region	NA	NA	Absent	n.a.	Novel
37	*MYH11*	c.5293C > T	p.(Arg1765Trp)	Missense	Coiled coil region	Zebrafish^b^	+ / + / + / 101	1 / 115948	n.a.	Novel
38	*NOTCH1*	c.2123A > G Mosaic	p.(Tyr708Cys)	Missense	EGF‐like 18	Tetraodon (FCUT Fruitfly)	+ / + / + / 194	Absent	*de novo* (inferred)	Novel
39	***PRKG1***	**c.530G > A**	**p.(Arg177Gln)**	**Missense**	**cGMP‐binding, high affinity**	***C. elegans*** b	**− / + / + / 43**	**Absent**	**n.a**.	**(** **Guo et al.,** 2013 **)**
40	***SLC2A10***	**c.510G > A** f	p.(Trp170*)	**Nonsense (NMD expected)**	**NA**	**NA**	**NA**	**Absent**	**n.a. (consaguineous parents)**	**(** **Coucke et al.,** 2006 **;** **Moceri et al.,** 2013 **)**
41	***SLC2A10***	**c.1276G > T** f	**p.(Gly426Trp)**	**Missense**	**Transmembrane helical region 10**	**Tetraodon** b	**+ / + / + / 184**	**3 / 116638**	**confirmed parental carriership**	**(** **Callewaert et al.,** 2008 **)**
42	***SMAD3***	**c.1A > T**	**p.(Met1?)**	**Loss of initiation codon**	**Initiator methionine**	***C. elegans*** b	**NA**	**Absent**	**n.a**.	**Novel**
43	***SMAD3***	**c.391_394dup**	p.(Thr132Argfs*35)	**Frameshift (NMD expected)**	**NA**	**NA**	**NA**	**Absent**	**n.a**.	**Novel**
44	***SMAD3***	**c.492dup**	p.(Asn165*)	**Frameshift (NMD expected)**	**NA**	**NA**	**NA**	**Absent**	**Yes**	**Novel**
45	*SMAD3*	c.802C > T	p.(Arg268Cys)	Missense	MH2	*C. elegans* b	+ / + / + / 180	Absent	Yes	Novel
46	*SMAD3*	c.893A > G	p.(Tyr298Cys)	Missense	MH2	Fruitfly	**−** / + / + / 194	Absent	Yes	Novel
47	***SMAD3***	**c.1010‐2A > G**	**p.?**	Splice (NMD not expected)	**MH2**	**NA**	**NA**	**Absent**	**n.a**.	**Novel**
48	***SMAD3***	**c.1179dup**	p.(Cys394Leufs*4)	**Frameshift (NMD not expected)**	**MH2**	**NA**	**NA**	**Absent**	**Yes**	**(** **Aubart et al.,** 2014 **)**
49	***TGFB2***	**c.709G > T**	p.(Glu237*)	**Nonsense (NMD expected)**	**NA**	**NA**	**NA**	**Absent**	**n.a**.	**Novel**
50	*TGFB2*	c.979C > T	p.(Arg327Trp)	Missense	Transforming growth factor beta‐2 chain	Frog	+ / + / + / 101	Absent	n.a.	(Lindsay et al., 2012; Schubert, Landis, Shikany, Hinton, & Ware, 2016)
51	*TGFB2*	c.989G > A	p.(Arg330His)	Missense	Transforming growth factor beta‐2 chain	Tetraodon	+ / + / + / 29	Absent	Incomplete penetrance?	Novel
**52** d	**TGFB2**	**c.1017‐1G > T**	**p.?**	**Splice (NMD possible)**	**Transforming growth factor beta‐2 chain**	**NA**	**NA**	**Absent**	***de novo***	**Novel**
**53**	***TGFB3***	**c.899G > A**	**p.(Arg300Gln)**	**Missense**	**Latency‐associated peptide chain**	**Fruitfly** b	**+ / + / + / 43**	**Absent**	**Yes**	**(Matyas, Naef, Tollens, & Oexle,** 2014 **)**
54	*TGFB3*	c.1075A > C	p.(Ser359Arg)	Missense	Transforming growth factor beta‐3 chain	Fruitfly^b^	+ / + / + / 110	Absent	n.a.	Novel
55	*TGFBR1*	c.790G > A	p.(Ala264Thr)	Missense	Protein kinase	Fruitfly^b^	+ / + / + / 58	Absent	yes (incomplete penetrance)	Novel
56	TGFBR1	c.1255+2T > C	p.[Tyr378Asnfs*3, 0]	Splice (exon 7 skipping partially stable at RNA level)	Protein kinase	NA	NA	Absent	yes	Novel
**57**	***TGFBR1***	**c.1460G > A**	**p.(Arg487Gln)**	**Missense**	**Protein kinase**	**Fruitfly** b	**+ / + / + / 43**	**Absent**	***de novo*** e	**(Akutsu et al.,** 2007 **; Jondeau et al.,** 2016 **; B. L. Loeys et al.,** 2006 **; Matyas et al.,** 2006 **; Melenovsky et al.,** 2008 **; Yang et al.,** 2012 **)**
58	*TGFBR2*	c.1565G > A	p.(Arg522Gln)	Missense	Protein kinase	Zebrafish^b^	+ / + / + / 43	1 / 121046	Paternally inherited	Novel
**59**	***TGFBR2***	**c.1630G > T**	p.(Glu544*)	**Nonsense (NMD not expected)**	**Protein kinase**	**NA**	**NA**	**Absent**	**n.a**.	**Novel**
**60**	***TGFBR2***	**c.1669C > T**	p.(Gln557*)	**Nonsense (NMD not expected)**	**Not in functional domain/region**	**NA**	**NA**	**Absent**	**n.a**.	**Novel**

Used RefSeq transcripts (based on Genome build: GRCh37/hg19): *ACTA2*: NC_000010.10(NM_001141945.2), *COL3A1*: NC_000002.11(NM_000090.3), *FBN1*: NC_000015.9(NM_000138.4), *FBN2*: NC_000005.9(NM_001999.3), *MYH11*: NC_000016.9(NM_001040113.1), *NOTCH1*: NC_000009.11(NM_017617.3), *PRKG1*: NC_000010.10(NM_001098512.2), *SLC2A10*: NC_000020.10(NM_030777.3), *SMAD3*: NC_000015.9(NM_005902.3), *TGFB2*: NC_000001.10(NM_001135599.2), *TGFB3*: NC_000014.8(NM_003239.4), *TGFBR1*: NC_000009.11(NM_004612.2), *TGFBR2*: NC_000003.11(NM_001024847.2).

Pathogenic variants (class 5) are depicted in bold.

FCUT, functionally conserved up to; n.a., not available; NA, not applicable; NMD, nonsense mediated mRNA decay

a Yes, segregation analysis performed in (at least) one family member, variant segregated accordingly.

b No further alignment available.

c This family is recently described in literature(Overwater & Houweling, 2017).

d A variant of unknown significance was identified in these patients as well (Supporting Information Table S1).

e Paternity and maternity not confirmed.

f Homozygous variant.

^‐^ Tolerated (SIFT), polymorphism (MutationTaster), and benign (Polyphen‐2) predictions.

^±^Possibly damaging (Polyphen‐2) prediction.

^+^Deleterious (SIFT), Disease‐causing (MutationTaster), probably damaging (Polyphen‐2) predictions.

Alignment, SIFT, MutationTaster, Polyphen‐2, Grantham distance: Alamut GRCh37 accessed July 2017.

**Table 2:** Summary of the clinical features of patient with a pathogenic or likely pathogenic variant detected by variant‐calling analysis of 21 H‐TAD genes

					Family history		
Patient	Involved gene	Sex, age^a^	Cardiovascular feature(s)	Systemic feature(s)	Genotype	Relative	Phenotype
1	*ACTA2*	♀, 16	PDA	None	+ − ? ?	F PU PA PGF	Dis (B, 51 y,), CVD Clinically not affected Dis, unconfirmed (deceased) Dis, unconfirmed (deceased)
2	*ACTA2*	♂, 28	Dis (A and B, 26 y), BAV	None	+^b^ −	F Sib	An (AoR 42 mm, AAo 49 mm, AA, 61 y) BAV Clinically not affected
3	*ACTA2*	♂, 46	Dis (A, 45 y)	None	?		No relatives clinically affected
4	*ACTA2*	♀, 69	Dis (B, 61 y; A, 65 y)	None	− + +	B (2) Si N	Clinically not affected Rup (AA, 62 y) An (AA, 35 mm)
5^c^	*ACTA2*	♂, 36	Dis (B, 36 y)	Iris flocculi, livedo reticularis	+ +	M D	Dis (B, deceased, 30 y), iris flocculi Iris flocculi
6	*ACTA2*	♂, 73	An (AoR, 52 mm, 69 y)	None	?		No relatives clinically affected
7	*ACTA2*	♂, 22	Dis (A, 21 y), BAV	PP, SS, Myopia −5/−5 dpt	?		No relatives clinically affected
8	*ACTA2*	♂, 57	Dis (B, 57 y), An (AoR 41 mm, 57 y)	Myopia −4 dpt, pneumothorax	?	B	SUD (58 y)
9^d^	*COL3A1*	♂, 59	Rup (AoA, 54 y), An (AA, 59 y)	None	? ? ?	B B N	Rup (AoA, deceased, 59 y) An (AA) An (AA, severe, 40 y)
10	*COL3A1*	♂, 52	Dis (A, 47 y), An (subclavian and vertebral artery, 52 y)	Increased AHR	?		No relatives clinically affected
11	*COL3A1*	♀, 44	Dis (B, 44 y)	NA	‐	Si	de novo^e^ Borderline An (AoR, 40 mm, 51 y), HT
12	*COL3A1*	♀, 31	An (renal and carotid artery), Dis (mammary‐, subclavian‐ and iliac artery), occlusion (brachial artery)	None	− ? +	F M Si	Clinically not affected Gastric perforation Dis (iliac artery)
13	*COL3A1*	♂, 42	Dis (A, 38 y)	Hyperkyphosis, hypermobile fingers	+ − −	M PU PGF	Clinically not affected Rup (AA, 55 y), CVD Rup (AA, 63 y), CVD
14	*COL3A1*	♂, 45	Dis (coronary artery, 42 y), An (AAo, 47 mm, 45 y)	Soft skin	?		No relatives clinically affected
15	*FBN1*	♂, 66	Dis (B, 49 y), An (subclavian artery, AA, 54 y)	NA	?	So	Clinical features of MFS
16	*FBN1*	♀, 27	An (AoR, 41 mm, 27 y), MVP	Arachnodactyly	? +	M D	Clinical features of MFS No clinical features of MFS (5 months)
17	*FBN1*	♂, 35	An (AoR, 50 mm, 35 y), ASD, atrial flutter (23 y)	Growth inhibiting treatment, HAP, crowding, retrognathia SS, IH	? ? ?	F PA PCo	SUD (44 y), clinical features of MFS SUD (43 y), clinical features of MFS Clinical features of MFS
18	*FBN1*	♂, 5	An (AAo, 27 mm, Z‐score +2.7, 5 y), VSD	PP, hyperkyphosis, wrist sign +, dolichocephaly, malar hypoplasia, EL, BS 8/9	?		No relatives clinically affected
19	*FBN1*	♂, 53	An (thoracic aorta, 80 mm, 53 y)	Wrist and thumb sign +, IH	?	PF	Multiple relatives with An and/or Dis
20	*FBN1*	♀, 36	An (AoR, severe, 35 y), MVP	Scoliosis, PC, Myopia −6.5 dpt, SS	?		No relatives clinically affected
21	*FBN1*	♂, 11	NA	Increased AHR, PD, clinical features of MFS			NA
22	*FBN1*	♂, 32	Dis (A, 15 y), MVP	Marfanoid habitus, PP, reduced elbow extension, arachnodactyly, HAP, crowding, myopia ‐5/−3 dpt, SS	?		No relatives clinically affected
23	*FBN1*	♀, 0	An (AoR,0 y), MI, TI	PC, joint contractures, arachnodactyly, dysmorphic facial features	?		No relatives clinically affected
24	*FBN1*	♂, 3	None	Height +3.4 SD, arachnodactyly, HAP, ptosis, epicanthal folds, delayed speech	+ ? ?	M MF MU	Arachnodactyly, tall stature Anamnestic MFS Premature birth, intracranial bleeding, epilepsy, spasticity, developmental delay
25	*FBN1*	♀, 29	An (AoR, 41 mm, 29 y), MI	Arachnodactyly, HAP, dolichocephaly, EL, RD	?	F	SD (42 y), myocardial infarction
26	*FBN1*	♀, 11	MVP	Marfanoid habitus, PP, wrist and thumb sign +, joint luxations, SS, recurrent hematomas	?		Clinically not affected
27	*FBN1*	♀, 9	None	Increased AHR, PC, club foot, PP, thumb sign +, downslanting, malar hypoplasia, myopia, recurrent hematomas	?		No relatives clinically affected
28	*FBN1*	♂, 5	None	Tall stature, arachnodactyly, PP, PC, wrist sign +, HAP, hypermobility, macular degeneration			*de novo* e
29	*FBN1*	♀, 10	An (AAo, 31 mm, Z‐score +2.7, 10 y)	PD, PP, arachnodactyly, HAP, dolichocephaly, myopia			*de novo* e
30	*FBN1*	♂, 54	Dis (A, 54 y)	Pneumothorax, NA	+	So(2)	Clinically not affected
31	*FBN1*	♀, 46	An (AAo, 46 mm, 46 y), cerebral infarction (33 y), stenosis (axillary‐, brachial‐ and subclavian artery, 36 y)	Hypermobile fingers	?		No relatives clinically affected
32	*FBN1*	♂, 0	MI, TI	PC, PP, dolichocephaly, downslanting, enophthalmos, floppy ears	?		No relatives clinically affected
33	*FBN2*	♂, 10	TI	Tall stature, PE, HAP, crowding	?	MF	An (aorta), hypermobility
34	*FBN2*	♂, 55	Borderline An (AAo, 39 mm, 54 y)	PE, hyperkyphosis, hammer toes, downslanting, myopia	+	F	Clinically not affected
35	*FBN2*	♂, 65	An (AAo, 45 mm, 64 y)	Hammer toes, HAP, enophthalmos, prominent eyes, and nose, malar hypoplasia	? −	F B	An (AA, at older age) An (AAo, 45 mm, 39 y)
36	*MYH11*	♂, 71	Dis (A and B, 70 y), An (AA, 54 mm, 71 y)	None	?	M	Rup (aorta, deceased)
37	*MYH11*	♂, 59	An (AAo, 46 mm, 58 y), BAV, PFO	PP, malar hypoplasia, cutaneous hyperextensibility	?		No relatives clinically affected
38	*NOTCH1*	♂, 77	An (AAo and AoA, 85 mm, 77 y)	None	?		*de novo* (inferred, mosaic) No relatives clinically affected
39	*PRKG1*	♂, 52	Dis (subclavian‐, iliac‐ and brachiocephalic artery, 42 y), borderline an (AAo, 40 mm, 52 y)	SS	?		No relatives clinically affected
40	*SLC2A10*	♀, 15	Arterial tortuosity (aorta, pulmonary artery, carotid arteries), MI, ASD	PP, hypermobile fingers, hypermobility, thumb sign +, clinodactyly, hypertelorism, periorbital fullness,	?		No relatives clinically affected
41	*SLC2A10*	♂, 0	An (AoR, 17 mm, Z‐score +3.3, 5 months), PFO, abnormal course AoA, and pulmonary vessels	Arachnodactyly, abnormal thumb position, downslanting, hypertelorism, HAP, retrognathia diaphragmatic hernia	HE HE	F M	Clinically not affected Clinically not affected
42	*SMAD3*	♀, 62	Dis (A, 60 y), MI	PP, early onset arthrosis, myopia −2.5/−4 dpt	?	F	An (AA, deceased, 67 y)
43	*SMAD3*	♂, 68	An (thoracic aorta)	Tall stature, PE, scoliosis, early onset arthrosis, mild myopia	+	D	Tall stature, arachnodactyly
44	*SMAD3*	♀, 37	Dis (coronary artery, 32 y), VSD	Brachydactyly type E, hypertelorism, prominent venous pattern, varicose veins, recurrent hematomas, myopia −6 dpt, IH, UH	? ?	M MGF	SUD (cause unknown, 50 y) SUD (cause unknown, 51 y)
45	*SMAD3*	♀, 76	Dis (B, 63 y), An (AoA, 60 mm, 70 y)	Arthralgia, genu valgum, hypermobility, IH	? + + +	So So GSo GDa	Dis (aorta, deceased, 44 y) Skeletal features fitting SMAD3 Borderline An (AoR, 40) Clinically not affected
46	*SMAD3*	♂, 17	None	Scoliosis, PE, flat cornea	+ ? ?	F PA PGM	An (cerebral, 49 y), PC SUD (anamnestic aneurysm AA, 40 y) SUD (anamnestic aneurysm AA, 60 y)
47	*SMAD3*	♀, 51	Dis (A, B, 51 y)	Scoliosis, arthralgia, early onset arthrosis	+	So	Clinically not affected
48	*SMAD3*	♀, 40	Borderline an (AoR, 40 y), MVP, MI	Wrist and thumb sign +, SS	+ ? ?	F PGM PF	Dis (A, 57 y), aneurysm (aorta, 40 y), HT Dis (thoracic aorta, 71 y) Several relatives with SUD (cause unknown)
49	*TGFB2*	♀, 19	None	Patellofemoral pain syndrome, wrist sign +, BS 7/9, downslanting, varicose veins	− −	M B	Clinically not affected Clinically not affected
50	*TGFB2*	♂, 39	An (AoR, 55 mm, 25 y), MVP	Scoliosis, PD, wrist and thumb sign +, hypermobility, recurrent hematomas in iliopsoas muscle, dural ectasia	?		No relatives clinically affected
51	*TGFB2*	♂, 0	None	Arachnodactyly, joint contractures, retrognathia	+ +	F PA	No clinical information available Dis (thoracic aorta)
52^d^	*TGFB2*	♂, 32	An (AoR, 44 mm, 32 y)	PC, PP, arachnodactyly, HAP, dolichocephaly, enophthalmos, malar hypoplasia, crowding, myopia −6.5 dpt, pneumothorax	− −	F B	de novo^e^ An (AAo, 52 mm, 65 y), BAV PD, PP, myopia
53	*TGFB3*	♂, 43	None	Increased AHR, PD, thumb sign +, BS 6/9	+ +	Si So	Clinical features of connective tissue disorder Clinical features of connective tissue disorder
54	*TGFB3*	♂, 59	AVI (25 y), An (AoR, 46 mm, 25 y; AoR, 55 mm, AAo 48 mm, 57 y)	PP, HAP, downslanting, UH	‐	So	Clinically not affected
55	*TGFBR1*	♂, 56	Dis (A and B, 56 y)	Scoliosis, PE, dolichocephaly, enophthalmos, malar hypoplasia	+	M	Clinically not affected
56	*TGFBR1*	♂, 33	An (AoR, 43 mm, 31 y)	SS, dural ectasia	+ + ?	M MA MGF	An (AoR, 44 mm, AAo, 44 mm, 58 y) An (thoracic aorta, 55 y) SUD (cause unknown, 64 y)
57	*TGFBR1*	♂, 16	Dis (thoracic aorta, deceased, 16 y)	PE, tall stature, scoliosis, arachnodactyly			*de novo*
58	*TGFBR2*	♂, 14	An (AoR, 40 mm, Z‐score +4.3, 14 y), VSD, DCRV	None	+	F	An (AoR, 42 mm, 52 y)
59	*TGFBR2*	♂, 15	None	PD, hyperkyphosis, arthralgia, myopia ‐3 dpt			NA
60	*TGFBR2*	♀, 16	An (AoR, 44 mm, 16 y), MVP	PP, arachnodactyly, hypermobility, luxations of hips and knees, bifid uvula, hypertelorism, blue sclerae			NA

AA, abdominal aortic; AAo, ascending aorta; AHR, arm / height ratio; An, aneurysm; AoA, aortic arch; AoR, aortic root; ASD, atrial septal defect; AVI, aortic valve insufficiency; B, brother; BAV, bicuspid aortic valve; BS, Beighton score; CVD, cardiovascular disease; D, daughter; DCRV, double chambered right ventricle; Dis, dissection; dpt, dioptre; EL, ectopia lentis; F, father; GDa, granddaughter; GSo, grandson; HAP, highly arched palate; HE, heterozygous carrier; HT, hypertension; IH, inguinal hernia; M, mother; MF, maternal family; MFS, Marfan syndrome; MGF, maternal grandfather; MI, mitral valve insufficiency; MU, maternal uncle; MVP, mitral valve prolapse; N, nephew; NA, no further information available; PA, paternal aunt; PC, pectus carinatum; PCo, paternal cousin; PD, pectus deformity; PDA, patent ductus arteriosus; PE, pectus excavatum; PF, paternal family; PFO, patent foramen ovale; PGF, paternal grandfather; PGM, paternal grandmother; PP, pes plani; PU, paternal uncle; RD, retinal detachment; Rup, rupture; SD, standard deviation; Si, sister; Sib, sibling; So, son; SS, skin striae; SUD, sudden death; TI, tricuspid valve insufficiency; UH, umbilical hernia; VSD, ventricular septal defect

a Age (in years) at DNA diagnostics.

b Low‐grade mosaicism detected by NGS analysis in the father of the index patient.

c This family is recently described in literature (Overwater & Houweling, 2017).

d A variant of unknown significance was identified in these patients as well (Supporting Information Table S1).

e Paternity and maternity not confirmed.

+ variant present

− variant absent

? unknown

### 3.2 Genetic and clinical data in patients with a CNV identified by XHMM analysis

The results of the XHMM analysis in the six patients with a CNV (patients 61–66) are depicted in Figure 1 and are summarized in Table 3.

Figure 1Genomic copy‐number variants in H‐TAD patients based on XHMM analysis. PCA: principal‐component analysis; XHMM: eXome hidden Markov model. **A,** *MYLK* gene; deletion of exons 17 and 18. **B,** *PRKG1* gene; deletion of exon 3. **C,** *SMAD3*; deletion of exon 6. **D,** *TGFB2*; deletion of exons 4, 5, 6, and 7. **E,** *NOTCH1* gene; whole gene duplication. **F,** *SCARF2* gene; whole gene deletion. Graphic representation of the copy‐number variants in each gene based on XHMM analysis. Horizontal axis indicates physical position of the CNVs. Vertical axis indicates sample Z‐score of PCA‐normalized read depth. Deletions are colored in red, and duplications are colored in green

**Figure:**
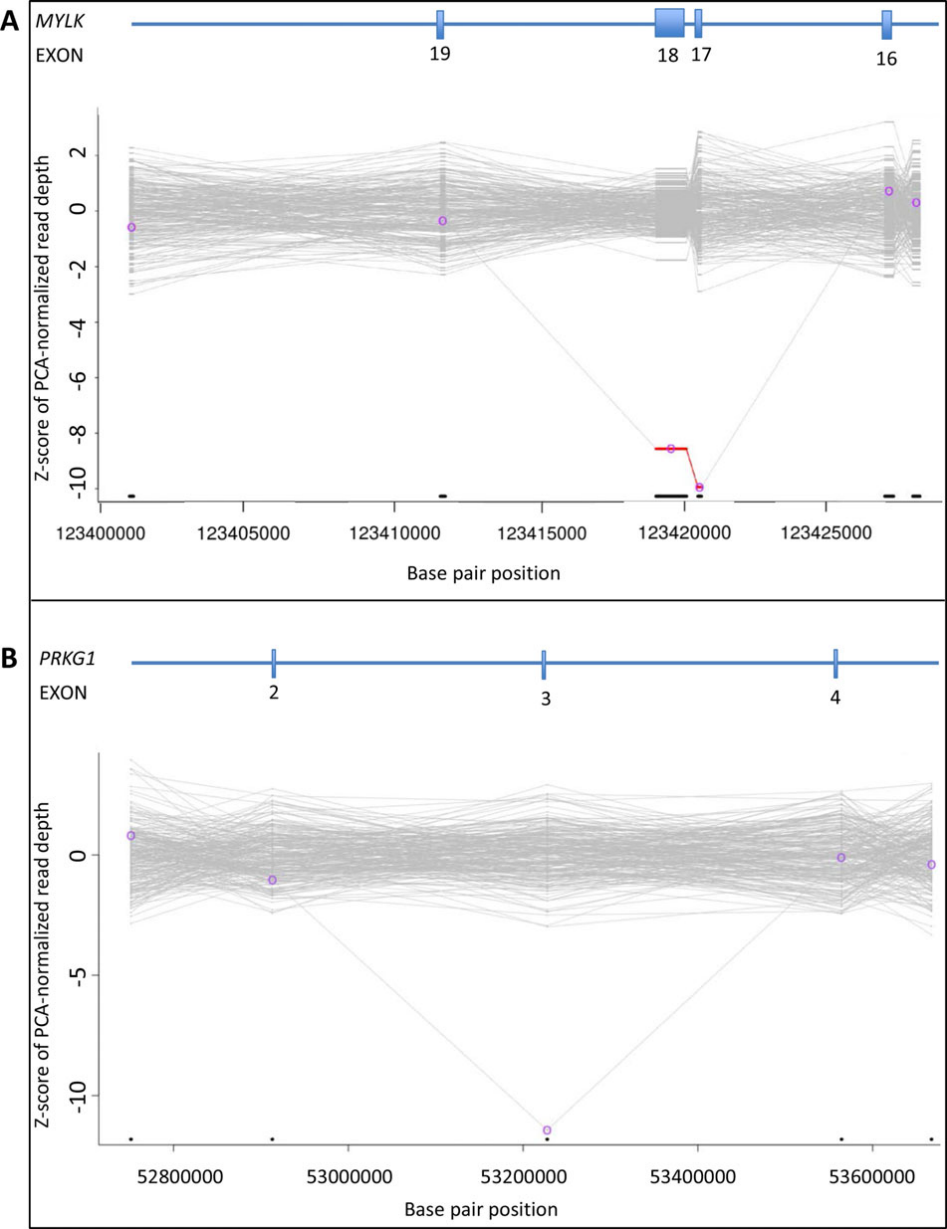

**Figure:**
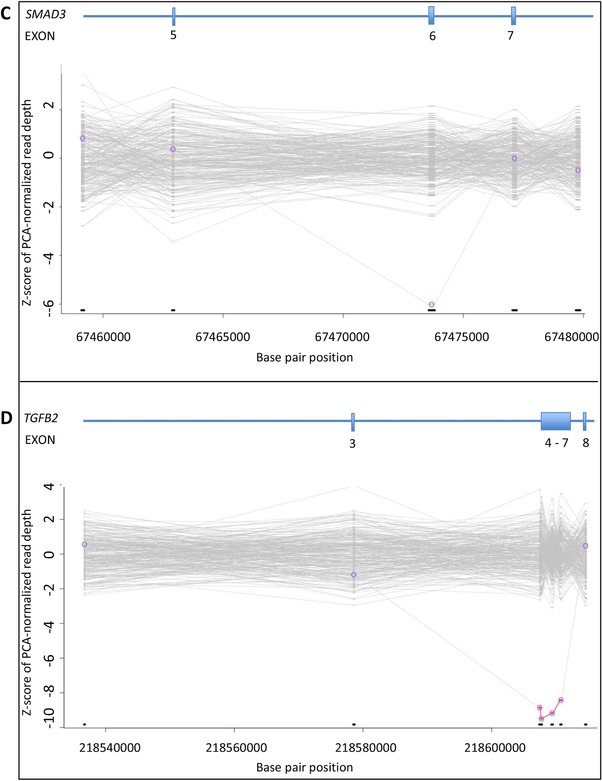

**Figure:**
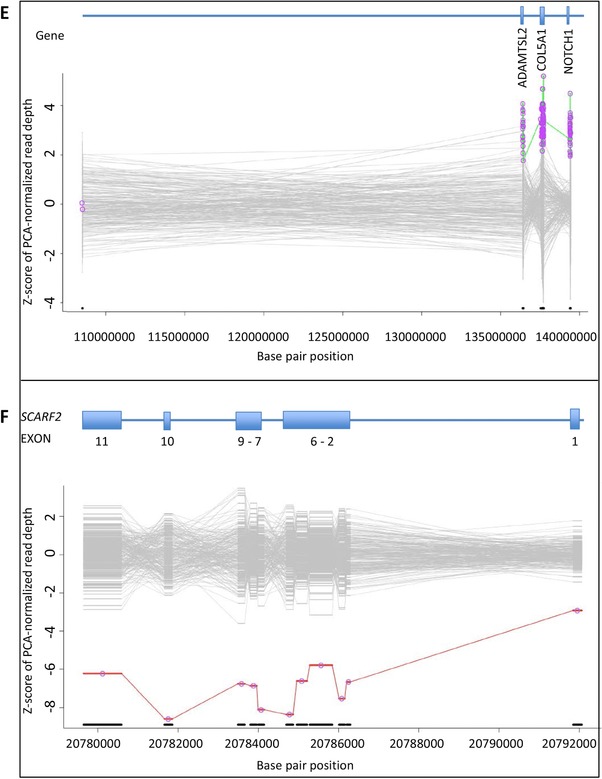

**Table 3:** Summary of the genetic features of six patients with a pathogenic or likely pathogenic CNV

Patient	Gender, age^a^	Involved gene, exon(s) based on XHMM analysis	Loss/gain	Protein change	Effect	Confirmed CNV	Validation technique	Variant classification
61	M, 66	*MYLK*; exon 17 and 18	Loss	Isoform 1 (NM_053025.3): p.(Asn798Leufs*13) Isoform 5 (smooth‐muscle cell specific): p.(0)	Frameshift (NMD expected) Loss of initiation codon (no protein expected)	*MYLK*, deletion exon 17 en 18^b^	MLPA	5
62	M, 36	*PRKG1*; exon 3^c^	Loss	p.(Asp145_Thr183delinsAla)	*in‐frame* deletion‐insertion	*PRKG1*, deletion exon 3^c^	MLPA	4
63	M, 31	*SMAD3*; exon 6^d^	Loss	p.(Asp220_Ile290del)	*in‐frame* deletion	*SMAD3*, deletion exon 6^d^	MLPA	5
64	M, 17	*TGFB2*; exons 4–7^e^	Loss	p.(Ile199_Arg390del)	*in‐frame* deletion	*TGFB2*, deletion exons 4–7^e^	MLPA	5
65	F, 0	Duplication *NOTCH1*; whole gene^f^	Gain	NA	NA	unbalanced translocation: 46,XX,der(7)t(7;9)(p22.3;q33.3)^f^	SNP array and karyotyping	5
66	M, 0	Deletion SCARF2; whole gene^g^	Loss	NA	NA	22q11.2 deletion: arr[hg19] 22q11.2(20779645_20792061)x1^g^	SNP array	5

CNV, copy‐number variation; MLPA, multiplex ligation‐dependent probe; NA, not applicable; NMD, nonsense mediated mRNA decay; XHMM, eXome hidden Markov model.

a Age (in years) at DNA diagnostics.

b HGVS nomenclature: NC_000003.11(NM_053025.3)(MYLK): c.(2390+1_2391‐1)_(3448+1_3449‐1)del.

c HGVS nomenclature: NC_000010.10(NssssssssM_001098512.2)(PRKG1): c.(433+1_434‐1)_(547+1_548‐1)del.

d HGVS nomenclature: NC_000015.9(NM_005902.3)(SMAD3): c.(658+1_659‐1)_(871+1_872‐1)del.

e HGVS nomenclature: NC_000001.10(NM_001135599.2)(TGFB2): c.(594+1_595‐1)_(1170+1_1171‐1)del.

f ISCN nomenclature after additional SNP array and karyotyping.

g ISCN nomenclature after additional SNP array.

In patient 61, a deletion of two exons in the *MYLK* gene was identified (NM_053025.3: c.(2390+1_2391‐1)_(3448+1_3449‐1)del). This deletion is predicted to generate an *out‐of‐frame* deletion in the long transcript of the *MYLK* gene (NM_053025.3) and a loss of the first 682 coding nucleotides, including the alternative translation initiation codon in the smooth‐muscle cell‐specific transcript encoding isoform 5 (Uniprot Q15746‐7). This male patient was diagnosed with a type B dissection at the age of 60 years and developed a type A dissection at the age of 65 years. He was treated surgically (Bentall procedure). Medical history and physical examination did not reveal any other signs of a connective tissue disorder. Pedigree analysis revealed that his sister suddenly died at the age of 53 years. No medical records, autopsy, or DNA were available. The 35‐year‐old son of the index patient did not carry the two‐exon deletion of *MYLK*. Until now, no other relatives opted for genetic testing.

In patient 62, a deletion of one exon of *PRKG1* was detected (NM_001098512.2: c.(433+1_434‐1)_(547+1_548‐1)del). This deletion is predicted to lead to an *in‐frame* deletion of 39 amino acids and the insertion of an Alanine residue and encompasses a large part of the high‐affinity cGMP‐binding domain of the PRKG1 protein including Arginine177. A recurrent substitution of this arginine for glutamine has been reported in patients with H‐TAD and shown to have a gain‐of‐function effect (Guo et al., 2013). At the age of 35 years, this male patient was diagnosed with an aortic root dilatation, a type A dissection, aortic valve insufficiency, and dilated cardiomyopathy. He was treated surgically (Bentall procedure). His skin showed stretch marks on the shoulders and chest. Medical history, ophthalmological evaluation, and physical examination did not reveal any other features of a connective tissue disorder. A cardiomyopathy gene panel analysis (50 genes) did not result in the identification of a genetic cause for his dilated cardiomyopathy. Family history showed no clinically affected relatives. No relatives were available for cardiologic evaluation and DNA diagnostics.

In patient 63, a deletion of one exon in *SMAD3*, predicted to result in an *in‐frame* deletion of part of the MH2 domain, was found (NM_005902.3: c.(658+1_659‐1)_(871+1_872‐1)del). This male patient was followed up from the age of eight years, after his father, who was diagnosed with a chronic dissection of the ascending aorta at the age of 33 years, suddenly died at the age of 37 years. The paternal grandmother died at the age of 39 years, possibly caused by an aortic dissection as well. The patient was diagnosed with an aortic root dilatation with a maximal diameter of 48 mm and a dilated left coronary artery at the age of 30 years. He was treated surgically (David procedure). Physical examination revealed pes plani, a prominent venous pattern on the chest and arms, and several dysmorphic facial features including dolichocephaly, hypertelorism, and retrognathia. He had no signs of early onset osteoarthritis.

In patient 64, a four‐exon deletion was detected in the *TGFB2* gene (NM_001135599.2: c.(594+1_595‐1)_(1170+1_1171‐1)del). This deletion is predicted to result in an *in‐frame* deletion of a large part of the TGFB2 protein. This 17‐year‐old male patient was under regular cardiologic surveillance because of TAAD in his father and paternal grandfather. At the age of 17 years cardiologic evaluation revealed an aortic root dilatation of 39 mm (Z‐score +3.28). Moreover, he had inguinal hernia repair at the age of one year, recurrent patellar dislocation, an asymmetric pectus deformity, and mild dysmorphic facial features including a long face, downslanting palpebral fissures, and a highly arched palate. The intragenic *TGFB2* deletion was also present in his clinically affected father (clinical features include aortic root aneurysm requiring surgery at age 31 and aortic dissection at age 46) and his 11‐year‐old sister (features consisted of pectus deformity and highly arched palate and mild myopia). The phenotypes of all family members will be described in more detail elsewhere (Vliegenthart et al., manuscript in preparation). All intragenic deletions were confirmed by MLPA analysis (Supporting Information Figure S1).

**Figure 2:**
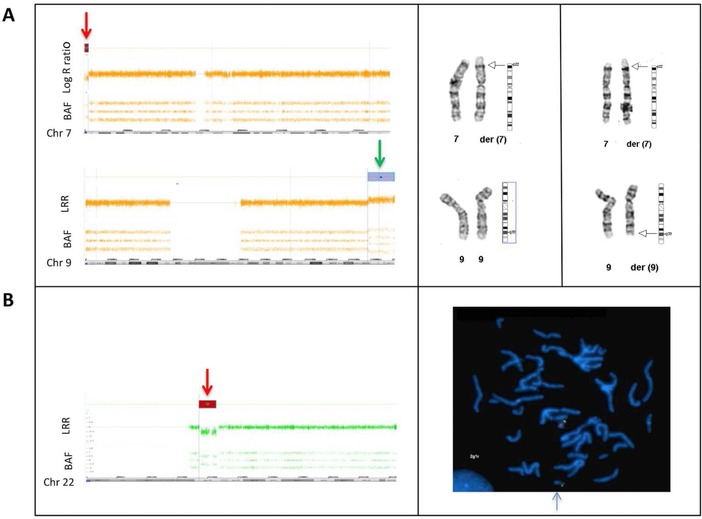
Further characterization of XHMM results by additional (cyto‐) genetic testing. BAF, B allele frequency; Chr, chromosome; der, derivate chromosome; LLR, log R ratio; FISH, fluorescence in situ hybridization. **A,** SNP array profile of chromosomes 7 and 9 are shown on the left. The top plot of each image shows the LRR, which provides an estimation of the copy number for each marker aligned to its chromosomal position. The bottom plot of each image shows the BAF for each SNP aligned to its chromosomal position. SNP array analysis revealed a terminal copy‐number loss at 7p22.3 (2Mb; GRCh37; chr7:43360‐2067625) indicated with a red arrow and a terminal copy‐number gain at 9q33.3–q34.43 (11.8Mb; GRCh37; chr9:129172353–141020389) indicated with a green arrow. Chromosomes 7 and 9 from the index (left) with the unbalanced translocation and the father (right) carrying the balanced translocation are shown on the right. The breakpoints of the reciprocal translocation are indicated with an arrow. The index has the derivative chromosome 7 lacking a short segment from the short arm of chromosome 7 that is replaced by an extra copy of a terminal segment of chromosome 9q. The father has two derivative chromosomes 7 and 9, each carrying a segment of the other chromosome. **B,** SNP array profile of chromosome 22 is shown on the left. SNP array analysis revealed a copy‐number loss at 22q11.2 (3.2Mb; GRCh37; chr22:20779645_20792061) indicated with a red arrow. The results of metaphase FISH on blood from the mother is presented on the right. The 22q11.2 region is recognized by the HIRA probe, producing a red signal. The green signal is from the ARSA probe hybridizing with the ARSA gene on chromosome band 22q13.33. The 22q11.2 deletion is indicated by a blue arrow. Metaphase FISH analysis revealed that the mother is also a carrier of the 22q11.2 deletion (ish del(22)(q11.2q11.2)(HIRA‐))

In patients 65 and 66, XHMM findings were suggestive of a larger chromosomal abnormality. In patient 65, a duplication of the entire *NOTCH1* gene was detected. *COL5A1* and *ADAMTSL2*, which are located in the same chromosomal region (9q) and are present in our NGS platform, were also duplicated in this newborn female patient who presented after birth with several dysmorphic features. Facial features included frontal bossing, deep‐set eyes, low set ears with overfolded helices, and a crumpled left ear with a preauricular tag, micrognathia, and a small mouth. In addition, flexion contractures of elbows, wrists, and knees and striking arachnodactyly were noticed. Based on these features, she was initially suspected to have neonatal Marfan syndrome or Beals syndrome. Because XHMM analysis indicated a large 9q duplication, an SNP array was performed. A copy‐number gain at 9q33.3–q34.43 (11.8Mb; hg19; chr9:129172353–141020389) and a copy‐number loss at 7p22.3 (2Mb; hg19; chr7:43360–2067625) were found. Subsequent karyotyping revealed an unbalanced translocation 46,XX,der(7)t(7;9)(p22.3;q33.3). Parental cytogenetic studies showed that her father carried a balanced reciprocal translocation; 46,XY,t(7;9)(p22.3;q33.3). Results of the array and karyotyping are shown in Figure 2A. In the literature, overlapping phenotypic manifestations such as similar craniofacial features, joint contractures, and arachnodactyly have been described in the 9q duplication syndrome (Amarillo, O'Connor, Lee, Willing, & Wambach, 2015). During follow‐up, she was treated for bleeding esophageal varices probably caused by portal vein thrombosis, which have not been described in patients with a 9q duplication syndrome and/or 7p22.3 deletion previously.

Finally, a deletion of the entire *SCARF2* gene, located at 22q11, was detected in patient 66. This newborn male patient presented with severe perinatal problems, including asphyxia and the need for resuscitation, after an uncomplicated pregnancy. Furthermore, initially a connective tissue disorder was suspected based on the presence of a relative dilatation of the aortic root in relation to the body surface area (16 mm, Z‐score +3) and a strangulated inguinal hernia. Physical examination revealed unilateral postaxial polydactyly without any other dysmorphic features. Simultaneous analysis of the NGS H‐TAD gene panel and SNP array revealed that the heterozygous deletion of *SCARF2* was part of a 22q11.2 deletion (i.e., DiGeorge syndrome) (3.2Mb; hg19; chr22:20779645_20792061). A normal male karyotype (46,XY) was seen. Parental fluorescence in situ hybridization (FISH) revealed that his mother also carried the 22q11.2 deletion (ish del(22)(q11.2q11.2)(HIRA‐)). Results of array and FISH are shown in Figure 2B. Except for delayed motor and speech development at childhood and complaints of fatigue and recurrent infections, his mother had no medical problems. Cardiac ultrasound showed no abnormalities. Most clinical features of the index patient, including inguinal hernia and postaxial polydactyly, were consistent with the established diagnosis. During follow‐up the relative dilatation of the aortic diameter was normalized.

## 4 DISCUSSION

This study provides the results of the molecular and clinical findings in the largest cohort of patients suspected of H‐TAD reported in the literature to date. In addition, this is the first report describing CNV analyses of 21 H‐TAD‐associated genes using variant‐calling analysis combined with XHMM analysis. In this cohort of 810 patients, a pathogenic or likely pathogenic variant was identified in 66 patients (8.1%). Overall, we identified a relatively low number of pathogenic or likely pathogenic variants in our H‐TAD cohort compared to previous studies that identified mutations in 10.3% to 35.5% (Campens et al., 2015; Lerner‐Ellis et al., 2014; Poninska et al., 2016; Proost et al., 2015; Wooderchak‐Donahue et al., 2015; Ziganshin et al., 2015). This wide range is likely to be explained by differences in clinical and demographic characteristics of the study populations and different inclusion criteria used for genetic testing. In general, DNA testing in the Netherlands is increasingly offered at a lower threshold to TAAD patients (e.g., not only to very young patients or patients with a positive family history for H‐TAD), which may explain the relatively low mutation detection yield.

Using routine NGS analysis (variant‐calling analysis) pathogenic or likely pathogenic variants were identified in *FBN1*, *ACTA2*, *SMAD3*, *COL3A1*, *TGFB2, TGFBR1*, *TGFBR2, FBN2*, *MYH11, TGFB3*, *SLC2A10*, *PRKG1*, and *NOTCH1*. As expected, most of the pathogenic and likely pathogenic variants were detected in *FBN1* (*N* = 18, 30%). Of these, at least 14 (78%) fulfilled the revised Marfan criteria. However, the proportion of pathogenic *FBN1* and *COL3A1* variants in this cohort is biased because single‐gene analysis of these two genes is still offered in our institute and variants in these genes detected using single‐gene analysis were not included in this study. Therefore, it is likely that in patients with a highly suggestive phenotype of vascular Ehlers–Danlos syndrome, single‐gene analysis of *COL3A1* was requested instead of NGS panel analysis. This might explain the high proportion of *COL3A1* variants predicted to result in haploinsufficiency detected in this study (3 of 6 = 50%, compared with approximately 4% of nonsense/frameshift variants currently reported in the COL3A1 LOVD database; https://eds.gene.le.ac.uk/home.php?select_db=COL3A1), as the phenotype in patients with COL3A1 haploinsufficiency is often confined to vascular events (Leistritz, Pepin, Schwarze, & Byers, 2011).

Of the pathogenic and likely pathogenic variants identified, 37 (67%) have not been described previously. None of these variants were identified more than once in our patient cohort. This emphasizes the extreme allelic heterogeneity of H‐TAD‐related disorders. Young age at diagnosis, a positive family history, and presence of syndromic features were shown to be the strongest predictors for the identification of a disease‐causing variant in the literature (*P* = 0.001–0.01) (Campens et al., 2015). The observation that the mean age at DNA testing in the group of patients with a pathogenic or likely pathogenic variant was 11 years lower than the mean age in the groups without a pathogenic or likely pathogenic variant is in line with this. However, 10 of the 66 patients with a pathogenic or likely pathogenic variant were over the age of 60 years at the time of DNA testing (15.2%). Of these, three patients (30%) had a negative family history for aortic disease, sudden death < 45 years, or systemic features of a connective tissue disorder. These observations underscore the reduced and age‐dependent penetrance with a high degree of clinical heterogeneity in H‐TAD. In five patients with an identified pathogenic or likely pathogenic variant, DNA testing of both parents suggested a *de novo* occurrence, while in one case a *de novo* occurrence was inferred as the variant was detected in mosaic status. This was in line with the negative family history for aortic disease in these families.

Of the 66 pathogenic or likely pathogenic variants, six were CNVs detected by XHMM analysis. These aberrations account for an incremental yield of 9.1% of the identified pathogenic or likely pathogenic variants, underscoring the relevance of adding a technique to identify CNVs in TAAD patients. The CNVs included (multi‐)exon deletions in *MYLK*, *PRKG1*, *SMAD3*, and *TGFB2*. To the best of our knowledge, intragenic (multi‐)exon deletions have not been reported in these genes before. The clinical features of the patients with these (multi‐)exon deletions did not differ notably from the known phenotypic manifestations related to variants in these genes. Moreover, a large duplication including the whole *NOTCH1* gene and a large deletion encompassing *SCARF2* were detected by XHMM analysis. These aberrations were part of an unbalanced translocation (46,XX,der(7)t(7;9)(p22.3;q33.3)) and a 22q11.2 deletion (22q11.2(20779645_20792061)x1), respectively, and were classified as the cause of the clinical features of the patients.

The results of this study underline the importance of CNV analysis using a bioinformatics tool such as XHMM in the clinical diagnostic care for TAAD patients. As CNV analysis is often not routinely performed for most genes included in this NGS platform, these CNVs would not have been detected by regular genetic analysis. Four of the six detected CNVs in this study were small intragenic deletions (two single‐exon deletions, one 2‐exon, and one 4‐exon deletion). These are generally not detected by routine CGH or SNP array analysis. This highlights the importance of using a CNV detection tool, which allows detection of CNVs with (small) single‐exon resolution. Based on the results of this study, single‐exon‐sensitive deletion/duplication analysis on a routine basis should be recommended in patients suspected of H‐TAD.

## 5 CONCLUSION

In 66 of 810 (8.1%) patients suspected of H‐TAD, a pathogenic or likely pathogenic variant was identified using our NGS gene panel in combination with XHMM analysis. Six of these 66 pathogenic or likely pathogenic variants (9.1%) were a CNV, not detectable by routine NGS analysis. This study is the first to describe the incremental yield of CNV analysis in patients suspected of H‐TAD. Our study underscores the importance of CNV analysis using a bioinformatics tool such as XHMM in the clinical diagnostic care for H‐TAD patients.

## Supporting information

Supplementary data: materials and methods
**Figure S1**. Confirmation of identified intragenic deletions with MLPA analysis
**Table S1**. Summary of the genetic features of patients with a variant of unknown significance detected by variant‐calling analysis of 21 H‐TAD genes
**Table S2**. Summary of the clinical features of the patient with a variant of unknown significance detected by variant‐calling analysis of 21 H‐TAD genes
**Table S3**. Overview of the genes analysed in this study
**Table S4**. Tools used to classify variants.
