# Supplementary material for: Results of next‐generation sequencing gene panel diagnostics including copy‐number variation analysis in 810 patients suspected of heritable thoracic aortic disorders
Source: Hum Mutat. 2018 Jul 12;39(9):1173–92. doi: 10.1002/humu.23565 (PMC6175145; doi:10.1002/humu.23565)
Supplement: Supplementary file 1 — Supplementary data: materials and methods Figure S1. Confirmation of identified intragenic deletions with MLPA analysis Table S1. Summary of the genetic features of patients with a variant of unknown significance detected by variant‐calling analysis of 21 H‐TAD genes Table S2. Summary of the clinical features of the patient with a variant of unknown significance detected by variant‐calling analysis of 21 H‐TAD genes Table S3. Overview of the genes analysed in this study Table S4. Tools used to classify variants. [file HUMU-39-1173-s001.docx]

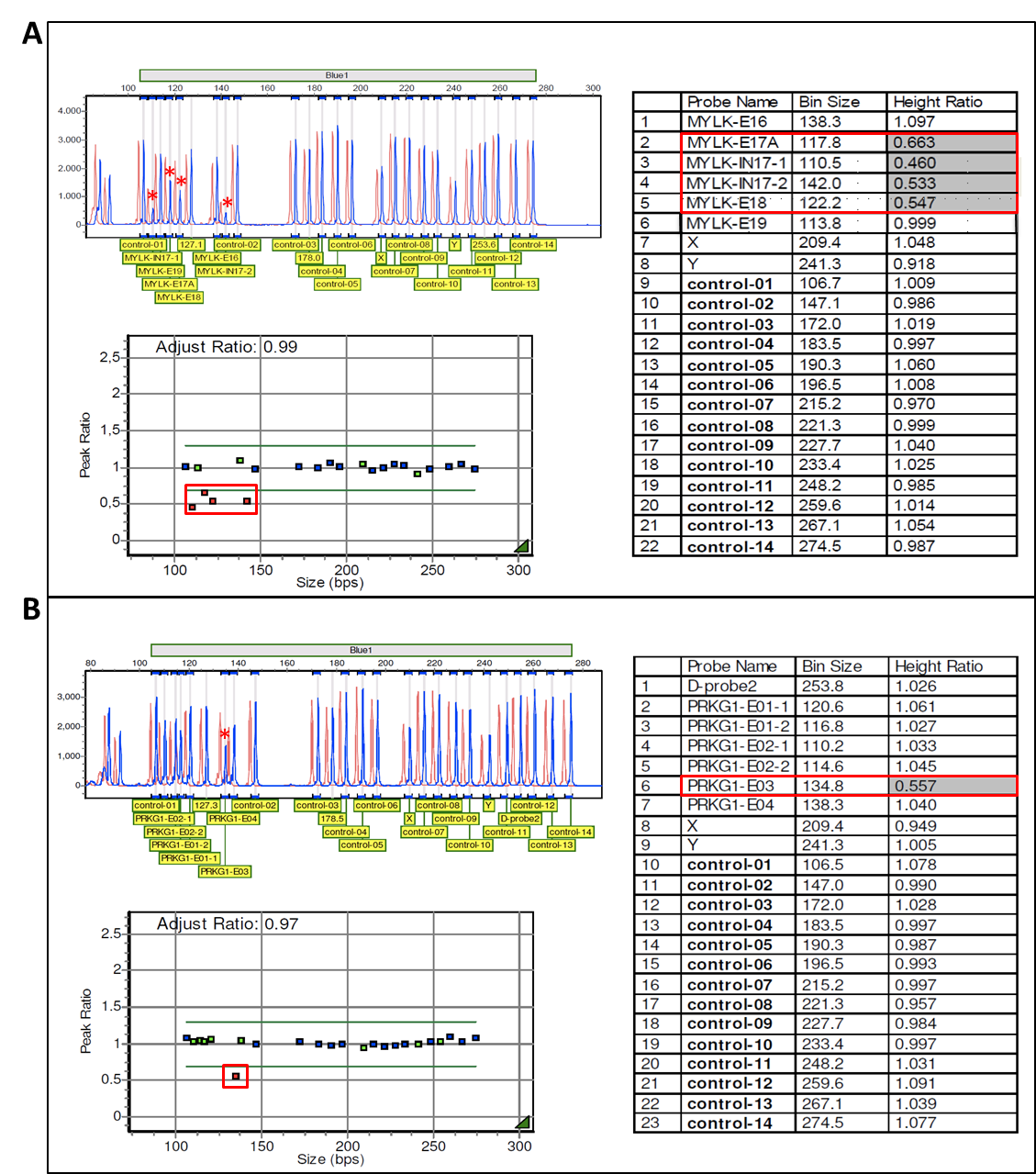


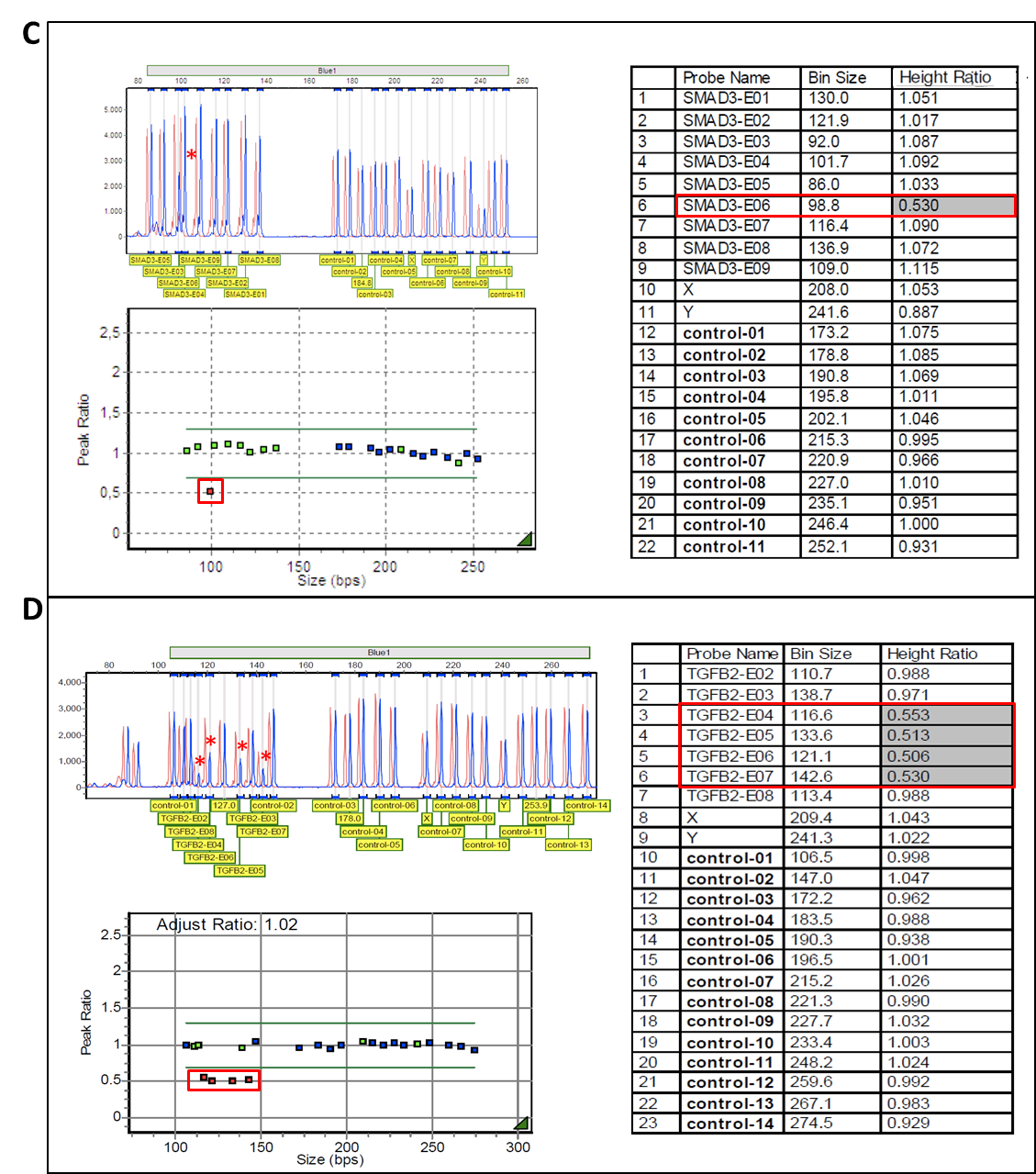


**Supplementary Figure S1. Confirmation of identified intragenic deletions with MLPA analysis**

MLPA: multiplex ligation-dependent probe amplification

**A** *MYLK* gene; deletion of exon 17 and 18.  **B** *PRKG1* gene; deletion of exon 3. **C** *SMAD3*; deletion of exon 6. **D** *TGFB2*; deletion exon 4, 5, 6 and 7.

For each index patient the GeneMarker electrophoretogram is shown in the upper left corner and the normalised MLPA data in the bottom left corner. The table on the right shows the ratio of signal from each MLPA probe compared to the combined normal reference control.

A height ratio of around 0.5 is suggestive of a deletion. Deletions are indicated with asterisks in the electrophoretogram and with a red square in the normalised MLPA data and in the table.

**Supplementary Table S1.** **Summary of the genetic features of patients with a variant of unknown significance detected by variant-calling analysis of 21 H-TAD genes**

| Patient ID | Gene | Nucleotide change | Protein change | Effect | Domain | | Conservation | | SIFT / MutationTaster / Polyphen-2 / Grantham distance | MAF ExAC | Segregation analysis^^^ | Reference |
| --- | --- | --- | --- | --- | --- | --- | --- | --- | --- | --- | --- | --- |
| 67 | *ACTA2* | c.13G>C | p.(Glu5Gln) | Missense | Actin | C. elegans | | - / + / - / 29 | | Absent | n.a. | Novel |
| 68 | *ACTA2* | c.607G>C | p.(Val203Leu) | Missense | Actin | Zebrafish | | - / + / - / 32 | | Absent | n.a. | Novel |
| 69^*^ | *COL3A1* | c.370G>C | p.(Gly124Arg) | Missense | N-terminal propeptide | Chicken^§^ | | + / + / + / 125 | | Absent | Yes | Novel |
| 70 | *COL3A1* | c.821A>G | p.(Glu274Gly) | Missense | Triple-helical region | Dog | | - / - / - / 98 | | Absent | n.a. | Novel |
| 71 | *COL3A1* | c.1472G>A | p.(Arg491Gln) | Missense | Triple-helical region | Chicken^§^ | | + / + / ± / 43 | | 2 / 121354 | Maternally inherited | Novel |
| 72 | *COL3A1* | c.1815+3del | p.? | Splice (NMD possible) | Triple-helical region | NA | | NA | | Absent | n.a. | Novel |
| 73 | *COL3A1* | c.2515C>T | p.(Pro839Ser) | Missense | Triple-helical region | Chicken^§^ | | + / + / - / 74 | | Absent | n.a. | Novel |
| 74 | *COL3A1* | c.2867G>A | p.(Arg956Gln) | Missense | Triple-helical region | Dog^§^ | | + / + / - / 43 | | Absent | n.a. | Novel |
| 75^*^ | *COL3A1* | c.4012T>A | p.(Phe1338Ile) | Missense | C-terminal propeptide | Chicken^§^ | | + / + / - / 21 | | Absent | n.a. | Novel |
| 76 | *ELN* | c.1150G>A | p.(Gly384Arg) | Missense | Ala-rich region | C. elegans^§^ | | + / + / + / 125 | | 4 / 121390 | n.a. | Novel |
| 77 | *ELN* | c.1150+1G>A | p.? | Splice (NMD not expected) | Ala-rich region | NA | | NA | | 6 / 121394 | n.a. | Novel |
| 78 | *ELN* | c.1489_1506del | p.(Leu497_Gly502del) | In-frame deletion | Ala-rich region | Moderately conserved residues | | NA | | 2 / 121356 | n.a. | Novel |
| 79 | *FBN1* | c.899A>G | p.(Glu300Gly) | Missense | EGF-like 5 | Chimp (FCUT Frog) | | - / - / - / 98 | | Absent | n.a. | Novel |
| 80 | *FBN1* | c.1577C>T | p.(Thr526Ile) | Missense | EGF-like 7 | Tetraodon^§^ | | + / + / ± / 89 | | Absent | n.a. | Novel |
| 81 | *FBN1* | c.1885G>A | p.(Val629Ile) | Missense | EGF-like 10 | Frog (FCUT Tetraodon^§^) | | - / + / - / 29 | | 1 / 121408 | n.a. | Novel |
| 82 | *FBN1* | c.2690G>A | p.(Gly897Asp) | Missense | TB 4 | Dog | | - / - / - / 94 | | Absent | n.a. | Novel |
| 75^*^ | *FBN1* | c.2979C>T | p.(Cys993=) | Silent | TB 5 | NA | | NA | | 2 / 121380 | n.a. | Novel |
| 83 | *FBN1* | c.4562C>T | p.(Pro1521Leu) | Missense | EGF-like 26 | Zebrafish^§^ | | + / + / + / 98 | | Absent | n.a. | Novel |
| 84 | *FBN1* | c.5363G>C | p.(Ser1788Thr) | Missense | EGF-like 29 | Zebrafish^§^ | | + / + / - / 58 | | Absent | n.a. | Novel |
| 52* | *FBN1* | c.5776A>G | p.(Asn1926Asp) | Missense | EGF-like 32 | Zebrafish^§^ | | + / + / ± / 23 | | Absent | n.a. | (Lebreiro et al., 2011) |
| 85 | *FBN1* | c.6055G>A | p.(Glu2019Lys) | Missense | EGF-like 35 | Frog | | + / + / ± / 56 | | Absent | Paternally inherited | (Sheikhzadeh et al., 2012; van de Luijtgaarden et al., 2015) |
| 86 | *FBN1* | c.6380-3A>G | p.? | Splice (NMD unlikely) | EGF-like 36 | NA | | NA | | 4 / 120530 | n.a. | (Baudhuin, Kotzer, & Lagerstedt, 2015) |
| 87 | *FBN1* | c.6449G>A | p.(Arg2150His) | Missense | EGF-like 36 | Zebrafish^§^ | | + / + / + / 29 | | Absent | Maternally inherited | Novel |
| 88 | *FBN1* | c.7064G>A | p.(Arg2355Lys) | Missense | TB 9 | Zebrafish^§^ | | - / + / - / 26 | | Absent | n.a. | Novel |
| 89 | *FBN1* | c.7241G>A | p.(Arg2414Gln) | Missense | EGF-like 41 | Frog (FCUT Zebrafish^§^) | | + / + / - / 43 | | 3 / 121272 | n.a. | (Sakai et al., 2006) |
| 90^*^ | *FBN1* | c.7379A>G | p.(Lys2460Arg) | Missense (effect on splicing excluded) | EGF-like 42 | Zebrafish^§^ | | + / + / + / 26 | | 9 / 121308 | n.a. | (Campens et al., 2015; Howarth et al., 2007; Robinson et al., 2012) |
| 91 | *FBN1* | c.7400A>G | p.(Gln2467Arg) | Missense | EGF-like 42 | Frog | | - / + / ± / 43 | | 3 / 121360 | n.a. | Novel |
| 92 | *FBN1* | c.7423A>G | p.(Ile2475Val) | Missense | EGF-like 42 | Tetraodon | | - / + / + / 29 | | Absent | n.a. | Novel |
| 93 | *FBN1* | c.7438G>A | p.(Gly2480Arg) | Missense | EGF-like 42 | Tetraodon | | + / + / + / 125 | | Absent | n.a. | Novel |
| 94 | *FBN1* | c.8232G>C | p.(Gln2744His) | Missense | Asprosin chain | Olive baboon | | - / - / - / 24 | | Absent | n.a. | Novel |
| 95 | *FBN2* | c.1423G>C | p.(Gly475Arg) | Missense | Not in functional domain/region | Chicken^§^ | | + / - / ± / 125 | | 3 / 121322 | n.a. | Novel |
| 96 | *FBN2* | c.1990A>T | p.(Thr664Ser) | Missense | EGF-like 10 | Chicken^§^ | | + / + / + / 58 | | Absent | n.a. | Novel |
| 97/98 | *FBN2* | c.2717G>A | p.(Arg906His) | Missense | TB 4 | Chicken^§^ | | + / + / - / 29 | | 14 / 121366 | n.a. | Novel |
| 99 | *FBN2* | c.3468C>T | p.(Cys1156=) | Silent (possible effect on splicing causing loss of a Cys) | EGF-like 16 | NA | | NA | | Absent | Maternally inherited | Novel |
| 100 | *FBN2* | c.4418G>A | p.(Arg1473His) | Missense | EGF-like 24 | Chicken^§^ | | + / + / + / 29 | | 1 / 120940 | n.a. | Novel |
| 101 | *FBN2* | c.5339C>G | p.(Pro1780Arg) | Missense | TB 7 | Chicken^§^ | | + / + / + / 103 | | Absent | yes | Novel |
| 90^*^ | *FBN2* | c.5635G>A | p.(Ala1879Thr) | Missense | EGF-like 30 | Chicken^§^ | | - / + / - / 58 | | Absent | n.a. | Novel |
| 102 | *FBN2* | c.6055G>A | p.(Glu2019Lys) | Missense | EGF-like 34 | Chicken^§^ | | - / + / - / 56 | | Absent | Maternally inherited | Novel |
| 103 | *FBN2* | c.6764A>G | p.(Asn2255Ser) | Missense | EGF-like 38 | Chicken^§^ | | + / + / + / 46 | | Absent | n.a. | Novel |
| 104 | *FBN2* | c.7723T>C | p.(Cys2575Arg) | Missense | EGF-like 44 | Chicken^§^ | | + / + / + / 180 | | Absent | n.a. | Novel |
| 105 | *MYH11* | c.161A>G | p.(Lys54Arg) | Missense | Not in functional domain/region | Chicken | | - / + / - / 26 | | Absent | n.a. | Novel |
| 106 | *MYH11* | c.1379C>G | p.(Ser460Cys) | Missense | Myosin motor | Zebrafish^§^ | | + / + / + / 112 | | Absent | Paternally inherited | Novel |
| 107 | *MYH11* | c.2216C>T | p.(Ala739Val) | Missense | Myosin motor | Zebrafish^§^ | | + / + / - / 64 | | 2 / 121388 | n.a. | Novel |
| 108 | *MYH11* | c.3583C>T | p.(Arg1195Trp) | Missense | Coiled coil | Zebrafish^§^ | | + / + / + / 101 | | 20 / 121406 | n.a. | Novel |
| 109/110 | *MYH11* | c.3787_3789del | p.(Lys1263del) | In-frame deletion | Coiled coil | Zebrafish^§^ | | NA | | 7 / 121398 | yes/yes | (Harakalova et al., 2013) |
| 111 | *MYH11* | c.4756G>A | p.(Asp1586Asn) | Missense | Coiled coil | Zebrafish^§^ | | + / + / + / 23 | | 6 / 121404 | n.a. | Novel |
| 9^*^ | *MYH11* | c.4870G>T | p.(Ala1624Ser) | Missense | Coiled coil | Zebrafish^§^ | | + / + / - / 99 | | Absent | n.a. | Novel |
| 112 | *MYH11* | c.4882A>C | p.(Lys1628Gln) | Missense | Coiled coil | Zebrafish^§^ | | + / + / ± / 53 | | 10 / 121268 | n.a. | Novel |
| 113 | *MYH11* | c.5267A>C | p.(Gln1756Pro) | Missense | Coiled coil | Zebrafish^§^ | | + / + / + / 76 | | Absent | n.a. | Novel |
| 114 | *MYLK* | c.575T>C | p.(Val192Ala) | Missense | Ig-like C2-type 2 | Chicken (FCUT Fruitfly^§^) | | + / + / + / 64 | | Absent | n.a. | Novel |
| 115 | *MYLK* | c.717G>A | p.(Ser239=) | Silent | Ig-like C2-type 2 | NA | | NA | | 2 / 121408 | n.a. | Novel |
| 116 | *MYLK* | c.1057C>A | p.(Gln353Lys) | Missense | Not in functional domain/region | Chicken | | + / - / - / 53 | | 1 / 121296 | n.a. | Novel |
| 117/118 | *MYLK* | c.2461C>T | p.(Arg821Trp) | Missense | Ig-like C2-type 6 | Chicken | | + / - / + / 101 | | 20 / 121160 | n.a. | Novel |
| 119 | *MYLK* | c.3121G>A | p.(Ala1041Thr) | Missense | 2-5 repeat | Dog | | - / - / - / 58 | | Absent | n.a. | Novel |
| 120 | *MYLK* | c.3610C>T | p.(Arg1204Trp) | Missense | Actin-binding (calcium/calmodulin-insensitive) region | Zebrafish | | + / + / ± / 101 | | 11 / 57930 | n.a. | Novel |
| 121 | *MYLK* | c.4222A>G | p.(Asn1408Asp) | Missense | Fibronectin type-III | Fruitfly | | - / + / + / 23 | | Absent | n.a. | Novel |
| 122 | *MYLK* | c.4565T>C | p.(Val1522Ala) | Missense | Protein kinase | Zebrafish (FCUT Baker’s yeast) | | + / + / ± / 64 | | 7 / 121338 | yes | Novel |
| 123 | *MYLK* | c.4850C>A | p.(Ser1617Tyr) | Missense | Protein kinase | Zebrafish | | + / + / + / 144 | | 1 / 121272 | n.a. | Novel |
| 69^*^ | *MYLK* | c.5477C>T | p.(Ala1826Val) | Missense | Ig-like C2-type 9 | Zebrafish | | - / + / + / 64 | | 34 / 121276 | yes | Novel |
| 124 | *NOTCH1* | c.631A>G | p.(Thr211Ala) | Missense | EGF-like 5 | Fruitfly^§^ | | + / + / - / 58 | | 3 / 18212 | n.a. | Novel |
| 125 | *NOTCH1* | c.1838G>A | p.(Arg613His) | Missense | EGF-like 16 | FCUT Zebrafish | | + / + / - / 29 | | 1 / 112762 | n.a. | Novel |
| 126 | *NOTCH1* | c.2542G>A | p.(Glu848Lys) | Missense | EGF-like 22 | Zebrafish | | + / + / + / 56 | | 319 / 118272 | Paternally inherited | Novel |
| 127^*^ | *NOTCH1* | c.2728G>A | p.(Asp910Asn) | Missense | EGF-like 24 | Fruitfly^§^ | | + / + / - / 23 | | 1 / 95336 | Yes, incomplete penetrance**^*^** | Novel |
| 128 | *NOTCH1* | c.3001G>A | p.(Gly1001Ser) | Missense | EGF-like 26 | Fruitfly^§^ | | + / + / + / 56 | | 2 / 21000 | n.a. | Novel |
| 129 | *NOTCH1* | c.3631C>T | p.(Arg1211Trp) | Missense | EGF-like 31 | Zebrafish | | + / + / + / 101 | | 3 / 117516 | n.a. | Novel |
| 130 | *NOTCH1* | c.4183A>G | p.(Asn1395Asp) | Missense | EGF-like 36 | Fruitfly^§^ | | - / + / - / 23 | | 2 / 102788 | n.a. | Novel |
| 131 | *NOTCH1* | c.6130G>A | p.(Ala2044Thr) | Missense | ANK 4 | Zebrafish | | + / + / + / 58 | | 6 / 120382 | n.a. | https://serval.unil.ch/resource/serval:BIB_F9BF67A18538.P001/REF |
| 132 | *NOTCH1* | c.6924C>T | p.(Cys2308=) | Silent (possible effect on splicing) | Not in functional domain/region | NA | | NA | | 1 / 117380 | n.a. | Novel |
| 133 | *PRKG1* | c.404A>C | p.(Asp135Ala) | Missense | cGMP-binding, high affinity region | Fruitfly (FCUT C. elegans^§^) | | + / + / - / 126 | | 1 / 120902 | n.a. | Novel |

| 134 | *PRKG1* | c.971T>C | p.(Leu324Ser) | Missense | cGMP-binding, low affinity | C. elegans^§^ | + / + / + / 145 | 3 / 118894 | n.a. | Novel |
| --- | --- | --- | --- | --- | --- | --- | --- | --- | --- | --- |

| 135 | *SMAD2* | c.448G>A | p.(Glu150Lys) | Missense | MH1 | Fruitfly | - / + / ± / 56 | Absent | Incomplete penetrance? | Novel |
| --- | --- | --- | --- | --- | --- | --- | --- | --- | --- | --- |
| 136 | *SMAD2* | c.711G>C | p.(Leu237Phe) | Missense | Not in functional domain/region | Frog | - / + / - / 22 | Absent | n.a. | Novel |
| 137 | *SMAD3* | c.394A>G | p.(Thr132Ala) | Missense | MH1 | Tetraodon | - / + / - / 58 | 3 / 121324 | n.a. | Novel |
| 138 | *SMAD3* | c.401T>C | p.(Val134Ala) | Missense | MH1 | Tetraodon (FCUT Fruitfly) | - / + / - / 64 | Absent | n.a. | Novel |
| 139 | *SMAD3* | c.785A>G | p.(Asp262Gly) | Missense | MH2 | Fruitfly | - / + / + / 94 | Absent | yes | Novel |
| 140 | *TGFB3* | c.196C>T | p.(Pro66Ser) | Missense | Latency-associated peptide | Fruitfly^§^ | + / + / ± / 74 | Absent | n.a. | Novel |
| 141 | *TGFB3* | c.389A>G  Mosaic | p.(Lys130Arg) | Missense | Latency-associated peptide | Zebrafish (FCUT Fruitfly^§^) | - / + / - / 26 | 2 / 121216 | *de novo* (inferred) | (Lidral et al., 1998) |
| 142 | *TGFB3* | c.412T>G | p.(Ser138Ala) | Missense | Latency-associated peptide | Chicken | - / + / - / 99 | 15 / 121256 | Maternally inherited | Novel |
| 143 | *TGFB3* | c.559G>A | p.(Gly187Ser) | Missense | Latency-associated peptide | Zebrafish (Ser in Olive baboon and Chicken) | - / - / - / 56 | 6 / 121378 | n.a. | (Ng et al., 2013) |
| 127^*^ | *TGFB3* | c.683C>T | p.(Pro228Leu) | Missense | Latency-associated peptide | Zebrafish | + / + / + / 98 | Absent | yes, incomplete penetrance**^*^** | Novel |
| 144 | *TGFB3* | c.797G>A | p.(Arg266His) | Missense | Latency-associated peptide | Zebrafish | - / + / - / 29 | 12 / 121408 | n.a. | Novel |
| 145 | *TGFB3* | c.873G>A | p.(Pro291=) | Silent (possible effect on splicing) | Latency-associated peptide | NA | NA | 13 / 121392 | n.a. | Novel |
| 146 | *TGFB3* | c.925C>T | p.(Arg309Cys) | Missense | Transforming growth factor beta-3 chain | Chicken | - / + / ± / 180 | Absent | no? | Novel |
| 147 | *TGFBR1* | c.134A>G | p.(Asn45Ser) | Missense | Not in functional domain/region | Fruitfly^§^ | + / + / + / 46 | 1 / 120998 | n.a. | (Goudie et al., 2011) |
| 148 | *TGFBR1* | c.923C>T  Mosaic | p.(Ser308Phe) | Missense | Protein kinase | Fruitfly^§^ | + / + / + / 155 | Absent | *de novo* (inferred) | Novel |
| 149 | *TGFBR1* | c.958A>G | p.(Ile320Val) | Missense | Protein kinase | Fruitfly^§^ | + / + / ± / 29 | Absent | yes | http://eprints.undip.ac.id/1097/1/Tesis_Nani.pdf |
| 150 | *TGFBR2* | c.1117C>T | p.(Arg373Cys) | Missense | Protein kinase | Dog | - / + / - / 180 | 3 / 119370 | n.a. | Novel |

Used RefSeq transcripts (based on Genome build: GRCh37/hg19): *ACTA2*: NC_000010.10(NM_001141945.2), *COL3A1*: NC_000002.11(NM_000090.3), *ELN*: NC_000007.13(NM_000501.3), *FBN1*: NC_000015.9(NM_000138.4), *FBN2*: NC_000005.9(NM_001999.3), *MYH11*: NC_000016.9(NM_001040113.1), *MYLK*: NC_000003.11(NM_053025.3), *NOTCH1*: NC_000009.11(NM_017617.3), *PRKG1*: NC_000010.10(NM_001098512.2), *SLC2A10*: NC_000020.10(NM_030777.3), *SMAD2*: NC_000018.9(NM_001003652.3), *SMAD3*: NC_000015.9(NM_005902.3), *TGFB2*: NC_000001.10(NM_001135599.2), *TGFB3*: NC_000014.8(NM_003239.4), *TGFBR1*: NC_000009.11(NM_004612.2), *TGFBR2*: NC_000003.11(NM_001024847.2).

FCUT, functionally conserved up to; n.a., not available; NA, not applicable; NMD, nonsense mediated mRNA decay

^ yes, segregation analysis performed in (at least) one family member, variant segregated accordingly

* Another variant was identified in these patients as well (see also Table 1)

§ No further alignment available

- Tolerated (SIFT), Polymorphism (MutationTaster), and Benign (Polyphen-2) predictions

± Possibly damaging (Polyphen-2) prediction

+ Deleterious (SIFT), Disease causing (MutationTaster), Probably damaging (Polyphen-2) predictions

Alignment, SIFT, MutationTaster, Polyp

**References**

Baudhuin, L. M., Kotzer, K. E., & Lagerstedt, S. A. (2015). Decreased frequency of FBN1 missense variants in Ghent criteria-positive Marfan

syndrome and characterization of novel FBN1 variants. *Journal of Human Genetics*, *60*(5), 241–252. <https://doi.org/10.1038/jhg.2015.10>

Campens, L., Callewaert, B., Mosquera, Muino, L., Renard, M., Symoens, S., De Paepe, … De Backer, J. (2015). Gene panel sequencing in heritable

thoracic aortic disorders and related entities - results of comprehensive testing in a cohort of 264 patients. *Orphanet Journal of Rare Diseases*, *10*,

9. <https://doi.org/10.1186/s13023-014-0221-6>

Goudie, D. R., D'Alessandro, M., Merriman, B., Lee, H., Szeverenyi, I., Avery, S., … Lane, E. B. (2011). Multiple self-healing squamous epithelioma is

caused by a disease-specific spectrum of mutations in TGFBR1. *Nature Genetics*, *43*(4), 365–369. https://doi.org/10.1038/ng.780

Harakalova, M., van der Smagt, J., de Kovel, C.G., Van't Slot, R., Poot,M., Nijman, I. J.,…Baas, A. F. (2013). Incomplete segregation of MYH11 variants

with thoracic aortic aneurysms and dissections and patent ductus arteriosus. *European Journal of Human Genetics*, *21*(5), 487–493. https://doi.org/10.1038/ejhg.2012.206

Howarth, R., Yearwood, C., & Harvey, J. F. (2007). Application of dHPLC for mutation detection of the fibrillin-1 gene for the diagnosis of Marfan

syndrome in a NationalHealth Service Laboratory. *Genetic Testing*, *11*(2), 146–152. https://doi.org/10.1089/gte.2006.0514

Lebreiro, A., Martins, E., Cruz, C., Almeida, J., Pimenta, S., Bernardes, M., … Abreu-Lima, C. (2011). [Genotypic characterization of a Portuguese population of Marfan

syndrome patients]. *Revista Portuguesa De Cardiologia*, *30*(7–8), 649–654. <https://doi.org/10.1016/S0870-2551(11)70003-8>

Lidral, A. C., Romitti, P. A., Basart, A. M., Doetschman, T., Leysens, N. J., Daack-Hirsch, S., … Murray, J. C. (1998). Association of MSX1 and TGFB3 with nonsyndromic clefting

in humans. *American Journal of Human Genetics*, *63*(2), 557–568. <https://doi.org/10.1086/301956>

Ng, D., Johnston, J. J., Teer, J. K., Singh, L. N., Peller, L. C., Wynter, J. S., … Program, N. I. H. I. S. C. C. S. (2013). Interpreting secondary cardiac disease variants in an exome

cohort. *Circulation. Cardiovascular Genetics*, *6*(4), 337–346. https://doi.org/10.1161/CIRCGENETICS.113.000039

Robinson, D. O., Lin, F., Lyon, M., Raponi, M., Cross, E., White, H. E., … Baralle, D. (2012). Systematic screening of FBN1 gene unclassified missense variants for splice

abnormalities. *Clinical Genetics*, *82*(3), 223–231. <https://doi.org/10.1111/j.1399-0004.2011.01781.x>

Sakai, H., Visser, R., Ikegawa, S., Ito, E., Numabe, H., Watanabe, Y., … Matsumoto, N. (2006). Comprehensive genetic analysis of relevant four genes in 49 patients with

Marfan syndrome or Marfan-related phenotypes. *American Journal of MedicalGenetics. Part A*, *140*(16), 1719–1725. <https://doi.org/10.1002/ajmg.a.31353>

Sheikhzadeh, S., Kade, C., Keyser, B., Stuhrmann, M., Arslan-Kirchner, M., Rybczynski, M., … von Kodolitsch, Y. (2012). Analysis of phenotype and genotype information for

the diagnosis of Marfan syndrome. *Clinical Genetics*, *82*(3), 240–247. https://doi.org/10.1111/ j.1399-0004.2011.01771.x

van de Luijtgaarden, K. M., Heijsman, D., Maugeri, A., Weiss, M. M., Verhagen, H. J., A, I. J., … Majoor-Krakauer, D. (2015). First genetic analysis of aneurysm genes in

familial and sporadic abdominal aortic aneurysm. *Human Genetics*, *134*(8), 881–893. https://doi.org/10.1007/s00439-015-1567-0

**Supplementary Table S2.** **Summary of the clinical features of the patient with a variant of unknown significance detected by variant-calling analysis of 21 H-TAD genes**

| **Patient** | **Involved gene** | **Sex, Age^#^** | **Cardiovascular feature(s)** | **Systemic feature(s)** | **Family history**  **(Genotype/Relative/Phenotype)** | | | | |  |
| --- | --- | --- | --- | --- | --- | --- | --- | --- | --- | --- |
| 67 | *ACTA2* | ♀, 65 | Dis (A, 42y) | PC, hindfoot deformity, malar hypoplasia, crowding, narrow face, RD | | ?  ? | B  Si | Tetralogy of Fallot  Borderline An (AAo, 40mm, 61y) |  |  |
| 68 | *ACTA2* | ♀, 43 | Dis (A, 42y), An (DAo, 53mm, 43y) | None | | ? | M | Dis (thoracic aorta, 46y) |  |  |
| 69* | *COL3A1* | ♂, 1 | Borderline An (AAo, 20mm, Z-score +2, 1y) | Plagiocephaly, metopic ridge | | +  -  ? | F  M  PGM | An (thoracic aorta, 49y)  Clinically not affected  SUD (cause unknown, 50y) |  |  |
| 70 | *COL3A1* | ♂, 65 | Dis (A, 65y), An (AA, 55mm, 64y) | PP | | ?  ?  ? | B  B  Si | An (thoracic aorta)  An (AA)  Dis (A) |  |  |
| 71 | *COL3A1* | ♀, 36 | Dis (carotid artery, 36y) | None | | -  + | F  M | Clinically not affected  Clinically not affected |  |  |
| 72 | *COL3A1* | ♂, 47 | Dis (carotid and vertebral artery, 38y, external iliac arteries, 46y), borderline An (AoR, 39mm, 46y) | None | | - | F | Clinically not affected |  |  |
| 73 | *COL3A1* | ♂, 55 | An (AoR 80mm, AAo 83mm, 55y) | Hindfoot deformity | | ?  ?  ? | MGF  MU  MCo | Dis (thoracic aorta, deceased, 72y)  An (84y)  An (57y) |  |  |
| 74 | *COL3A1* | ♂, 63 | Dis (A, 62y) | None | | ? |  | Clinically not affected |  |  |
| 75* | *COL3A1* | ♂, 45 | An (AoR 59mm, AAo 42mm, 44y) | PP, hip dysplasia | | ? | PF | Multiple relatives with An (thoracic aorta) |  |  |
| 76 | *ELN* | ♀, 50 | An (AoR, 43mm, 48y) | PE, hindfoot deformity, retrognathia, malar hypoplasia | | ?  ? | F  B | An (AA, deceased, 78y), CVD  An (AA, deceased, 39y), CVD |  |  |
| 77 | *ELN* | ♂, 50 | An (DAo, 48mm, 50y) | None | | ? |  | Clinically not affected |  |  |
| 78 | *ELN* | ♂, 41 | An (AAo, 46mm, 41y), BAV | None | | ? |  | Clinically not affected |  |  |
| 79 | *FBN1* | ♂, 66 | An (AAo, 49mm, 65y) | Recurrent ankle joint luxations, HAP, blue sclerae, IH | | ?  ? | M  B | Dis (aorta)  Dis (thoracic aorta, deceased, 69y) |  |  |
| 80 | *FBN1* | ♂, 47 | An (AoR, 56mm, AAo, 40mm, 47y), MVP, AF | Downslanting, iris diaphany, varicose veins, dural ectasia, IH | | +  +  + | Si  N  N | Clinically not affected  An (AoR, 53mm, 45y)  An (AoR, 46mm,43 y) |  |  |
| 81 | *FBN1* | ♀, 55 | An (AAo, 50mm, 55y), BAV | Pneumothorax, pelvic organ prolaps | | ? | FN | An (aorta) |  |  |
| 82 | *FBN1* | ♂, 14 | An (AAo, 30mm, Z-score +3.1, 14y), BAV | HAP | | ? |  | Clinically not affected |  |  |
| 75* | *FBN1* | ♂, 45 | An (AoR 59mm, AAo 42mm, 44y) | PP, hip dysplasia | | ? | PF | Multiple relatives with An (thoracic aorta) |  |  |
| 83 | *FBN1* | ♂, 30 | An (AoR, 54mm, 29y) | PC, PP, arachnodactyly, HAP, dolichocephaly, enophthalmos, malar hypoplasia, crowding, myopia -6.5 dpt, pneumothorax | | ?  ? | F  PF | SUD (cause unknown, 57y)  Anamnestic MFS |  |  |
| 84 | *FBN1* | ♂, 41 | An (AoR, 48mm, AAo, 53mm, 41y) | Myopia -7/-8 dpt, SS | | ? |  | Clinically not affected |  |  |
| 52* | *FBN1* | ♂, 32 | An (AoR, 44mm, 32y) | PC, PP, arachnodactyly, HAP, dolichocephaly, enophthalmos, malar hypoplasia, crowding, myopia -6.5 dpt, pneumothorax | | +  - | F  B | An (AAo, 52mm, 65y), BAV  PD, PP, myopia |  |  |
| 85 | *FBN1* | ♀, 10 | Borderline An (AoR, 25mm, Z-score +2, 10y), MVP | Tall stature, increased intraocular pressure | | +  ? | F  PA | NA  SUD (cause unknown, 38y) |  |  |
| 86 | *FBN1* | ♀, 40 | Dis (B, 39y) | None | | + | M | NA |  |  |
| 87 | *FBN1* | ♂, 8 | None | Arachnodactyly, increased AHR, PD, wrist and thumb sign +, BS 8/9, hypermobile fingers, retrognathia, downslanting | | + | M | Clinically not affected |  |  |
| 88^a^ | *FBN1* | ♀, 49 | PDA | Scoliosis, height and OFC ≥3SD, craniofacial features fitting Sotos syndrome, myopia -3.75/-6 dpt | | ? |  | Clinically not affected |  |  |
| 89 | *FBN1* | ♂, 14 | None | Scoliosis, arachnodactyly, marfanoid habitus, camptodactyly, craniosynostosis, pes equinus, HAP, malar hypoplasia, crowding, long face, developmental delay, anxiety disorder | | ? |  | Clinically not affected |  |  |
| 90* | *FBN1* | ♂, 37 | Dis (A, 37), BAV | Scoliosis | | ? | So | Tall stature, scoliosis, SS, myopia |  |  |
| 91 | *FBN1* | ♂, 49 | An (AoR, 48mm, 49y), DCM, AF | None | | ? |  | Clinically not affected |  |  |
| 92 | *FBN1* | ♀, 50 | An (AoR, 47mm, AAo, 43mm, 50y) | None | | ? | So | BAV and VSD |  |  |
| 93 | *FBN1* | ♂, 45 | An (AoR 48mm, AAo 42mm, 45y), BAV | None | | ? | PCo (3) | BAV |  |  |
| 94 | *FBN1* | ♂, 48 | Dis (carotid artery, 29y), borderline An (AoR, 40mm, 48y) | Pneumothorax | | ? | F | SUD (CVD, 40y) |  |  |
| 95 | *FBN2* | ♀, 32 | Dis (coronary artery, 32y) | None | | ? |  | Clinically not affected |  |  |
| 96 | *FBN2* | ♀, 46 | Dis (A, 46y) | NA | | ? | PA | Dis (thoracic aorta) |  |  |
| 97 | *FBN2* | ♂, 55 | Dis (B, 52y), An (AA) | Myopia -4.5 dpt | | ? |  | Clinically not affected |  |  |
| 98 | *FBN2* | ♂, 54 | An (AoR, 44mm, AAo, 46mm, 53y) | Glaucoma | | ? | B | An (AoR, 51mm) |  |  |
| 99 | *FBN2* | ♂, 14 | An (AoR, 33mm, Z-score +3.4, AAo, 27mm, 14y) | Scoliosis, PP, arachnodactyly, wrist sign +, pes valgus, genu valgus, SS | | + | M | Clinically not affected |  |  |
| 100 | *FBN2* | ♀, 41 | Borderline An (AAo, 40mm, 41y) | Increased AHR | | ?  ? | M  Si | Dis (thoracic aorta, deceased, 50y)  Dis (thoracic aorta, deceased, 46y) |  |  |
| 101 | *FBN2* | ♀, 63 | An (AAo, 42mm, 60y) | Downslanting, retrognathia, HAP | | +  ? | MA  MCo | An (AoR and AAo, 76y)  Dis (36y) |  |  |
| 90* | *FBN2* | ♂, 37 | Dis (A, 37), BAV | Scoliosis | | ? | So | Tall stature, scoliosis, SS, myopia |  |  |
| 102 | *FBN2* | ♀, 13 | NA | Tall stature, arachnodactyly, clinodactyly | | + | M | Clinically not affected |  |  |
| 103 | *FBN2* | ♂, 64 | An (AA) | NA | |  |  | NA |  |  |
| 104 | *FBN2* | ♀, 58 | An (AAo, 43mm, 57y), BAV | None | | ? | B | BAV |  |  |
| 105 | *MYH11* | ♀, 46 | An (AAo 47mm, 45y), BAV | None | | ? | F | An (aorta) |  |  |
| 106 | *MYH11* | ♂, 0 | An (AoR, 15mm, Z-score +3.7, 2 months) | None | | +  ? | F  PF | Clinically not affected  SUD (cause unknown) |  |  |
| 107 | *MYH11* | ♀, 68 | An (AAo, 45mm, 68y) | Scoliosis, hyperkyphosis, varicose veins, UH | | ?  ?  ? | F  B  Si | SUD (cardiac arrest, 55y)  Dis (thoracic aorta, 64y)  SUD (no aortopathy, 46y) |  |  |
| 108 | *MYH11* | ♂, 47 | An (AoR, 42mm, 47y) | SS, varicose veins | | ?  ?  ? | M  MU  MA | An (aorta, 52mm)  Dis (thoracic aorta, deceased, 71y)  Dis (thoracic aorta, 55y) |  |  |
| 109 | *MYH11* | ♂, 47 | Dis (B, 33y), An (AAo, 80mm, 33y and DAo, 66mm, 47y) | Scoliosis, PE | | + | So | Dis (A, 17y), BAV |  |  |
| 110 | *MYH11* | ♀,40 | NA | NA | |  |  | NA |  |  |
| 111 | *MYH11* | ♂, 74 | An (AA, 38mm, 74y), CVD | None | |  |  | NA |  |  |
| 9* | *MYH11* | ♂, 59 | Rup (AoA, 54y), An (AA, 59y) | None | | ?  ?  ? | B  B  N | Rup (AoA, deceased, 59y)  An (AA)  An (AA, severe, 40y) |  |  |
| 112 | *MYH11* | ♀, 52 | An (AoA) | NA | |  |  | NA |  |  |
| 113 | *MYH11* | ♂, 17 | An (AoR, 35mm, Z-score +2.77, 17y), BAV | SS, IH | | ? |  | Clinically not affected |  |  |
| 114 | *MYLK* | ♀, 56 | An (AAo, severe, 55y) | Myopia -4 dpt | | ? |  | Clinically not affected |  |  |
| 115 | *MYLK* | ♂, 58 | An (AoR, 48mm, 58y) | PE, HAP, myopia -6 / -4 dpt | | ? |  | Clinically not affected |  |  |
| 116 | *MYLK* | ♂, 53 | Diss, subarachnoid hemorrhage | NA | |  |  | NA |  |  |
| 117 | *MYLK* | ♀, 43 | An (AoR, 42mm, AAo, 43mm, 43y), ASD, PDA | BS 5/9 | | ?  ?  ? | F  M  PCo | Dis (iliac arteries)  Dis (A, B, 66y)  PDA |  |  |
| 118 | *MYLK* | ♂, 7 | None | PE, prominent venous pattern, retrognathia, malar hypoplasia, HAP | | ? |  | Clinically not affected |  |  |
| 119 | *MYLK* | ♂, 50 | An (AoR and AAo, 60mm, 49y), BAV | None | | ? |  | Clinically not affected |  |  |
| 120 | *MYLK* | ♂, 58 | An (AoR, 45mm, AAo, 43mm, 58y) | None | | ?  ? | So  So | WPW (deceased, 14y)  WPW |  |  |
| 121 | *MYLK* | ♀, 38 | Dis (vertebral artery, 38y) | None | | ? |  | Clinically not affected |  |  |
| 122 | *MYLK* | ♂, 62 | An (AoR, 48mm, AAo 40mm, 62y), RBBB | Increased AHR, PP, myopia -2.75 / -3.75 dpt, glaucoma | | +  ? | B  B | An (AAo, 43mm, 42y), RBBB  Borderline An (AoR, 40mm) |  |  |
| 123 | *MYLK* | ♀, 15 | Borderline An (AoR, 31mm, Z-score +1.7, 15y) | Scoliosis, PE, SS, retrognathia, malar hypoplasia | | ? |  | Clinically not affected, |  |  |
| 69* | *MYLK* | ♂, 1 | Borderline An (AAo, 20mm, Z-score +2, 1y) | Plagiocephaly, metopic ridge | | +  -  ? | F  M  PGM | An (thoracic aorta, 49y)  Clinically not affected  SUD (cause unknown, 50y) |  |  |
| 124 | *NOTCH1* | ♂, 57 | Dis (A, 57y), BAV | None | | ? |  | Clinically not affected |  |  |
| 125 | *NOTCH1* | ♂, 62 | An (AoR, 46mm, AAo, 48mm, 62y) | None | | ? |  | Clinically not affected |  |  |
| 126 | *NOTCH1* | ♀, 9 | An (AAo, 36mm, Z-score +6.7, 9y), BAV, valvular aortic stenosis | None | | + | F | NA |  |  |
| 127* | *NOTCH1* | ♂, 73 | An (AoR, 53mm, AAo, 46mm, AoA 42mm, 74y) | Increased AHR, scoliosis, early onset arthrosis, HAP, varicose veins, IH | | +  ?  +  +  ?  ?  ? | Si  Si  So  So  N  Co  Co | An (AoA), Dis (DAo, deceased, 67y)  Dis (DAo, deceased, 64y)  An (AoA, 47mm, 49y)  An (AoA, 42mm, 47y)  An (AoA, severe, 36y), Dis (cerebral An, deceased, 53y)  Borderline An (thoracic aorta)  BAV |  |  |
| 128 | *NOTCH1* | ♂, 69 | An (AoR and AAo, 70mm, 62y) | None | | ?  –  ? | F  B  So | Dis (aorta, deceased, 67y)  BAV (deceased, 47y)  Dis (carotid artery) |  |  |
| 129 | *NOTCH1* | ♀, 69 | Dis (A, 69y) | NA | | ?  ?  ?  ? | B  Si  C  PF | Dis (B, deceased, 46y)  Borderline An (74y)  An (AA, 60y)  Anamnestic aortic Diss |  |  |
| 130 | *NOTCH1* | ♀, 65 | Dis (B, 64y), An (AAo, 46mm, AA, 64mm, 64y), MI, AVI | IH | | ? | F | SUD (CVD, deceased 53y) |  |  |
| 131 | *NOTCH1* | ♀, 78 | An (AAo, 51mm, 78y), AF | NA | |  |  | NA |  |  |
| 132 | *NOTCH1* | ♂, 64 | Dis (A, 39y) | None | | ? |  | Clinically not affected |  |  |
| 133 | *PRKG1* | ♂, 69 | An (AoR, 43mm, 51y), MI | None | | ? |  | Clinically not affected |  |  |
| 134 | PRKG1 | ♂, 48 | Dis (several aortic branches) | NA |  | |  | NA | | |
| 135 | *SMAD2* | ♂, 13y | An (AAo, 24mm, Z-score +2, 13y) | PE, scoliosis, wrist sign +, pes valgus, genu valgus, malar hypoplasia, HAP | | + | F | HAP, IH |  |  |
| 136 | *SMAD2* | ♂, 65 | An (AA, 59mm, 65y) | None | | ? |  | Clinically not affected |  |  |
| 137 | *SMAD3* | ♂, 66 | An (AoR, 51mm, AAo 52mm, 66y) | IH | | ? |  | Clinically not affected |  |  |
| 138 | *SMAD3* | ♂, 39 | An (AoR 44mm, AAo, 42mm, 38y) | None | | ? | F | SUD (cause unknown, 52y) |  |  |
| 139 | *SMAD3* | ♀, 53 | Dis (A, 52y) | Scoliosis, PE, hindfoot deformity, spondylolisthesis, increased AHR, HAP, downslanting palpebral fissure, malar hypoplasia | | -  +  +  - | Sib  So  So  So | Clinically not affected  An (AoR, 39mm, Z-score +3.7, 17y), increased AHR, hindfoot deformity, hyperkyphosis, retrognathia, enophthalmos, downslanting  PP  Clinically not affected |  |  |
| 140 | *TGFB3* | ♂, 63 | An (splenic and pancreaticoduedenal artery, 61y) | None | | ? | Si | SUD (intracranial hemorrhage, 29y) |  |  |
| 141 | *TGFB3* | ♂, 68 | An (AA, 57mm, common iliac artery, 24mm, 68y) | None | | ? |  | Clinically not affected |  |  |
| 142 | *TGFB3* | ♀, 35 | Dis (A, 35y) | None | | ? |  | Clinically not affected |  |  |
| 143 | *TGFB3* | ♀, 72 | An (AAo, 43mm, 68y), AF | Hyperkyphosis | | ? | C | An (thoracic aorta) |  |  |
| 127* | *TGFB3* | ♂, 73 | An (AoR, 53mm, AAo, 46mm, AoA 42mm, 74y) | Increased AHR, scoliosis, early onset arthrosis, HAP, varicose veins, IH | | +  ?  +  +  +  +  ?  - | Si  Si  So  So  N  Co  Co  Co | An (AoA), Dis (DAo, deceased, 67y)  Dis (DAo, deceased, 64y)  An (AoA, 47mm, 49y)  An (AoA, 42mm, 47y)  An (AoA, severe, 36y), Dis (cerebral An, deceased, 53y)  Borderline An (thoracic aorta), clubfeet  BAV  Clinically not affected |  |  |
| 144 | *TGFB3* | ♂, 38 | An (AoR, 42mm, 38y) | SS, PD | | ? |  | Clinically not affected |  |  |
| 145 | *TGFB3* | ♂, 68 | An (AoR, 42mm, 68y), MVP, TI, AF | Increased AHR, Scoliosis, PP, HAP, dolichocephaly, malar hypoplasia, varicose veins, IH, UH | | ?  ?  ? | M  B  B | MVP  SUD (cause unknown, 41y)  SUD (cause unknown, 55y) |  |  |
| 146 | *TGFB3* | ♂, 65 | An (AAo, 48mm, 64y), BAV | None | | - | So | Borderline An (AoR) |  |  |
| 147 | *TGFBR1* | ♀, 52 | Dis (A, 52y), An (AoR, 43mm, AAo, 47mm, 48y, iliac-, femoral-, visceral- and pulmonary artery) | Hypermobility | | ?  ?  ? | F  M  MF | SUD (cause unknown, 59y)  SUD (cause unknown, 65y)  Multiple relatives with An (thoracic aorta) and/or dis |  |  |
| 148 | *TGFBR1* | ♀, 28 | None | BS 5/9, recurrent preterm delivery | | -  - | F  So | An (aorta)  Clinically not affected |  |  |
| 149 | *TGFBR1* | ♂, 37 | Borderline An (AoR, 39mm, 37y) | Increased AHR, PD, malar hypoplasia, myopia -3 dpt, prominent venous pattern | | ?  ? | F  PU | Dis (aorta, 47y)  SUD (cause unknown, 51y) |  |  |
| 150 | *TGFBR2* | ♂, | Dis (A, 52y), ASD | Arachnodactyly, HAP, long face | | ? | F | SUD (cause unknown, 52y) |  |  |

AA, abdominal aortic; AAo, ascending aorta; AF, atrial fibrillation; AHR, arm / height ratio; An, aneurysm; AoA, aortic arch; AoR, aortic root; ASD, atrial septal defect; AVI, aortic valve insufficiency; B, brother; BAV, bicuspid aortic valve; BS, Beighton score; CVD, cardiovascular disease; DAo, descending aorta; DCM, dilated cardiomyopathy; Dis, dissection; dpt, dioptre; F, father; HAP, highly arched palate; IH, inguinal hernia; M, mother; MA, maternal aunt; MCo, maternal cousin; MF, maternal family; MFS, marfan syndrome; MGF, maternal grandfather; MI, mitral valve insufficiency; MU, maternal uncle; MVP, mitral valve prolapse; N, nephew; NA, no further information available; OFC, occipitofrontal circumference; PA, paternal aunt; PC, pectus carinatum; PCo, paternal cousin; PD, pectus deformity; PDA, patent ductus arteriosus; PE, pectus excavatum; PF, paternal family; PGM, paternal grandmother; PP, pes plani; PU, paternal uncle; RBBB, right bundle branch block; RD, retinal detachment; Rup, rupture; SD, standard deviation; Si, sister; Sib, siblings; So, son; SS, skin striae; SUD, sudden death; TI, tricuspid valve insufficiency; UH, umbilical hernia; VSD, ventricular septal defect; WPW, Wolff-Parkinson White syndrome

^#^ Age (in years) at DNA diagnostics

* Another variant was identified in these patients as well (Table 1 and Supp. Table S1)

+ variant present

- variant absent

? unknown

^a^ A likely pathogenic *NSD1* variant was detected (Sotos syndrome)

**Supplementary data: materials and methods**

**Samples**

DNA was isolated from peripheral blood leucocytes using an automated DNA isolator (Chemagen MSM1/MICROLAB STAR Autoload system, Chemagen, Perkin Elmer, Waltham, MA, USA/Hamilton Robotics, Reno, NV, USA).

**NGS targeted gene panel testing**

All patients underwent targeted NGS analysis using a platform enriched to allow diagnostic testing of genes involved in several connective tissue disorders (CTD, version 2, 88 genes). The SeqCap EZ Choice Library kit (IRN4000018830, Nimblegen) was used to design a custom-based platform enriched in the selected connective tissue genes. With this platform, the enrichment is achieved with an ‘in solution’ methodology, using baits. Sequence analysis was performed using MiSeq Reagent Kit v3 (150 cycle) (Illumina, San Diego, California, USA) for a PE75 run on a MiSeq sequencing system (Illumina). For most of the genes analyzed in this study, a coverage of at least 30 times was reached for the entire targeted region (Supplementary table 1). The average sequencing depth was 282 reads. A few genes presented in some of the patients with one or more low-coverage region. Low-coverage regions in “core” genes were analyzed by Sanger sequencing, using standard protocols (Supp. Table S3).

**Variant-calling analysis**

For the bioinformatics analysis, an in-house diagnostic pipeline was used. Alignment of the reads was performed by Burrows-Wheeler Aligner (BWA; v0.7.8) (Li & Durbin, 2010). Post-processing of the bam files and variant-calling analysis was done using the Genome Analysis Tool Kit Lite (v3.3-0-g37228af) (McKenna et al., 2010). Picard Tools (v1.111, http://picard.sourceforge.net) and Samtools (v01.18) (Li et al., 2009) were used to convert formats and remove duplicates. Selection of the genes of interest (Supp. Table S3) and analysis of the data was performed using Cartagenia (www.cartagenia.com, Leuven, BE). The variants in the region of interest (i.e. exonic and +/-20 nucleotides in the intron) were filtered using a curated in-house database.

**CNV detection analysis**

Deletion/duplication analysis was performed for all the genes included in the targeted NGS platform using XHMM (eXome hidden Markov Model), a bio-informatics read-depth based tool for the detection of copy number variations in NGS data (<https://atgu.mgh.harvard.edu/xhmm/>). Before implementation in diagnostics, we validated XHMM on our targeted connective tissue disorders (CTD) platform. For this validation, a “training-set” of 1210 samples was used to determine the optimal settings for XHMM. These samples had been earlier analyzed using a previous version of our CTD platform (version 1, 48 CTD-associated genes included). Moreover, for most of these samples MLPA data of either *FBN1*, *TGFBR2*, *COL3A1*, *TNXB* and *PLOD1* or *COL5A1*, *COL3A1*, *TNXB* and *PLOD1* or *COL1A1* and *COL1A2* were available (SALSA MLPA kits P065, P066, P155, P271, P272, P331, P332, P359, MRC-Holland). A “test-set” of 318 samples, analyzed with CTD platform version 2 (88 CTD-associated genes) and MLPA, was successively used to verify that the “optimal” XHMM settings were also applicable to CTD platform version 2. From this test-set, a “clean” control set of 241 samples was created by removing every sample that contained either a depth of coverage outlier or a CNV. Every time a new sample is tested with XHMM, it will be run together with the control set in order to see if a called CNV is significant or not. Among the samples which were excluded from the test-set, 35 samples with previously (by MLPA or SNP-array) detected CNVs were present, which were all detected by XHMM. Detected CNVs included, among others, 12 single-exon deletions or duplications and larger CNVs as 3 whole-gene deletions or duplications (AM&MMW, unpublished data).

For the intragenic CNVs found in this study (patients 61-64), confirmation of XHMM results was performed by multiplex ligation-dependent probe amplification (MLPA) analysis. In all these cases, gene-specific home-designed probes were added to either the SALSA MLPA P300 or P200 probemix (MRC Holland, Amsterdam, NL). The test was successively performed according to the instructions of the supplier. Both large CNVs detected by XHMM in patients 65 en 66, were confirmed by the results of follow-up analysis performed by SNP array and (for patient 65) karyotyping.

# Variant classification

Variants were classified based on assessment of the available evidence, in line with the guidelines of the ACMG Laboratory Practice Committee Working Group (Richards et al., 2015), integrated with our professional expertise and knowledge of the genes and disorders we routinely offer diagnostic service for.

Each variant is individually assessed based on the following evidence/criteria:

a) Gene and disease information

- disease pathogenetic mechanism

- mode of inheritance

- disease incidence

- penetrance and expression variability

- phenotype match and phenotype specificity

- gene constraint (tolerance for variations)

b) Variant information

- loss of function (LOF)

- change in physical and chemical properties (including polarity, charge, size)

- amino acid conservation across species

- localization in functional domain or motif

- effect on molecule processing, amino acid (post-translational) modification, and protein-protein interaction

- in silico analysis predictions

c) Database and literature information

- observation of the same variant in public and internal databases (both population databases or patients/locus specific databases) and in literature

- observation of similar (in terms of localization and/or disease mechanism) variants in public and internal databases (both population databases and patients/locus specific databases) and in literature

d) segregation analysis (de novo assessment, segregation with the disease phenotype)

e) functional studies

A list of tools routinely used for variant classification is reported in Supplementary Table S4.

All variants are classified into one of the five following categories: pathogenic, likely pathogenic, benign, likely benign, or uncertain significance.

# Pathogenic variant:

- Variant predicted to result in the loss of protein function in a gene for which this is a known mechanism of disease. This applies only to some of the genes included in this study, namely *COL3A1, EFEMP2, FBN1, MYLK, NOTCH1, PLOD1, SCARF2, SLC2A10, SMAD3, SMAD4, TGFB2, TGFB3.*
- Known/recurrent disease causing variant, based on evidence in the literature: variant is reported in multiple affected (unrelated) individuals, phenotype of the reported patients fits with the assessed associated genetic disorder, variant segregates with the disorder.
- Variant demonstrated to result in loss/reduced or aberrant protein function in an appropriate functional assay, consistently with the pathogenetic mechanism of the disorder.
- Variant predicted (position -1, -2, +1, +2) or demonstrated (all other exonic or intronic positions) to cause aberrant splicing in an appropriate functional assay, and result in a unstable or aberrant RNA, in line with the established pathogenetic mechanism of the disorder.
- Type of variant for which a pathogenetic mechanism is known and well established in literature (e.g.: substitution of a Glycine residu in the triple helix domain of collagen type III or substitution or introduction of a Cysteine residu in a EGF-like domain of fibrillin 1).
- *de novo* presentation, in combination with strong molecular, functional or database/literature evidences of pathogenicity.

**Likely pathogenic variant:**

- LOF variant in gene for which LOF pathogenetic mechanism has not been established but is expected.
- Variant for which all the following conditions apply :
  - variant affects a highly conserved amino acid
  - variant affects a highly conserved region, functional domain or motif, or is predicted to affect amino acid (post-translational) modification
  - variant has not been detected or is rare in control individuals (allele frequency compatible with mode of inheritance, incidence and penetrance of the disorder)
  - variant is reported (though in a limited number of cases) in literature, phenotype of the reported patients fits with the assessed associated genetic disorder. Or: variant has not been previously reported in literature but similar variants (same localization/domain/mechanism) have been previously reported and demonstrated to be pathogenic and the gene/domain shows low tolerance for variations
  - segregation data (when available) are consistent with co-segregation with the disorder (taken in account penetrance/age of onset)
  - expected effect is consistent with pathogenetic mechanism of disorder

**Likely benign**

One of more of the following conditions apply:

- Variant found in (multiple) unaffected individuals at an allele frequency inconsistent with a clinical significance of the variant, based on mode of inheritance, penetrance and severity of disorder.
- Silent or intronic variant with in silico prediction not suggestive of an effect on splicing.
- Lack of segregation with disease within a family (taking into account penetrance, age of onset, phenocopies).
- Conservative change of non-conserved amino acid.
- Functional evidence or other evidence (e.g. localization of the variant) suggests no deleterious effect of the variant.
- Inconsistence with established pathogenetic mechanism of disorder.

**Benign**

- Variant reported in control populations at a frequency undoubtedly inconsistent with a clinical significance, based on mode of inheritance, penetrance and severity of disorder.
- Other well established evidence that the variant has no clinical significance.

**Variant of Uncertain clinical Significance (VUS):**

- Insufficient or conflicting evidence to establish pathogenicity or benign effect of a variant.

**Supplementary Table S3. Overview of the genes analyzed in this study**

| **Gene** | **Genomic sequence** | **Reference sequence** | **Chromosomal location** | **Average coverage (%)** | **Exons with a low quality XHMM profile** |
| --- | --- | --- | --- | --- | --- |
| *ACTA2* | NC_000010.10 | NM_001141945.2 | 10q23.31 | 100 | - |
| BGN^#^ | NC_000023.10 | NM_001711.4 | Xq28 | 100 | - |
| *COL3A1* | NC_000002.11 | NM_000090.3 | 2q32.2 | 100 | - |
| *EFEMP2* | NC_000011.9 | NM_016938.4 | 11q13 | 100 | - |
| *ELN* | NC_000007.13 | NM_000501.3 | 7q11.1-q21.1 | 100 | - |
| *FBN1* | NC_000015.9 | NM_000138.4 | 15q21.1 | 100 | - |
| *FBN2* | NC_000005.9 | NM_001999.3 | 5q23-q31 | 100 | - |
| *MYH11* | NC_000016.9 | NM_001040113.1 | 16p13.11 | 100 | - |
| *MYLK* | NC_000003.11 | NM_053025.3 | 3q21 | 100 | - |
| *NOTCH1* | NC_000009.11 | NM_017617.3 | 9q34.3 | >99* | Exon 1 |
| *PLOD1* | NC_000001.10 | NM_000302.3 | 1p36.22 | 100 | - |
| *PRKG1* | NC_000010.10 | NM_001098512.2 | 10q11.2 | 100 | - |
| SCARF2^$^ | NC_000022.10 | NM_153334.6 | 22q11.21 | >98 | Exon 1, 4, 11A |
| *SKI* | NC_000001.10 | NM_003036.3 | 1p36.33 | >99 | Exon 7 |
| *SLC2A10* | NC_000020.10 | NM_030777.3 | 20q13.12 | 100 | Exon 1 |
| *SMAD2* | NC_000018.9 | NM_001003652.3 | 18q21 | 100 | - |
| *SMAD3* | NC_000015.9 | NM_005902.3 | 15q21-q22 | >99* | - |
| *SMAD4* | NC_000018.9 | NM_005359.5 | 18q21.1 | 100 | - |
| *TGFB2* | NC_000001.10 | NM_001135599.2 | 1q41 | 100 | - |
| *TGFB3* | NC_000014.8 | NM_003239.2 | 14q24 | 100 | - |
| *TGFBR1* | NC_000009.11 | NM_004612.2 | 9q22 | >99* | Exon 1 |
| *TGFBR2* | NC_000003.11 | NM_001024847.2 | 3p22 | 100 | - |

^#^Included in the gene panel since October 2016, analyzed in 166 patients included in this study.

^$^Excluded from the gene panel since October 2016, analyzed in all but 166 patients included in this study.

*Exons with a coverage of less than 30 reads in the targeted NGS analysis were successively sequenced with Sanger sequencing.

**Supplementary table S4. Tools used to classify variants.**

| **Tool** | **Reference** | **Purpose** |
| --- | --- | --- |
| Alamut® Visual 2.9.0 | http://www.interactive-biosoftware.com/alamut-visual/ | This program integrates and/or facilitates access to variant prediction tools, evolutionary amino acid conservation data, protein domains data, variant databases. |
| *SIFT, Version 6.2.1 | Ng and Henikoff. SIFT: predicting amino acid changes that affect protein function. Nucleic Acids Research, 2003, Vol. 31, No. 13 3812-3814 | Variant prediction method.  Variants are predicted to be:  - Tolerated  - NOT tolerated (= Deleterious) |
| *MutationTaster2 | Schwarz et al. MutationTaster2: mutation prediction for the deep-sequencing age.  Nat Methods. 2014 Apr;11(4):361-2. | Variant prediction method.  Variants are predicted to be:   - Disease causing - Polymorphism |
| PolyPhen-2.2.2 (build 398)  (HumVar-trained model) | Adzhubei et al. A method and server for predicting damaging missense mutations. Nat Methods 7(4):248-249 (2010) | Variant prediction method.  Variants are predicted to be:   - Probably damaging - Possibly damaging - Benign |
| *Splice Site Finder-like | Zhang et al. [Statistical features of human exons and their flanking regions](https://www.ncbi.nlm.nih.gov/pubmed/9536098). Hum Mol Genet (1998) vol. 7 (5) pp. 919-32. | Splice site prediction method^#^ |
| *MaxEntScan  (Maximum Entropy Model) | Yeo and Burge. Maximum entropy modeling of short sequence motifs with applications to RNA splicing signals [J Comput Biol.](https://www.ncbi.nlm.nih.gov/pubmed/15285897) 2004;11(2-3):377-94 2004 | Splice site prediction method^#^ |
| *NNSPLICE, version 0.9 | Reese et al. [Improved splice site detection in Genie.](https://www.ncbi.nlm.nih.gov/pubmed/9278062) J Comput Biol. 1997 Fall;4(3):311-23. | Splice site prediction method^#^ |
| *Gene Splicer | Pertea et al. GeneSplicer: a new computational method for splice site prediction. [Nucleic Acids Res.](https://www.ncbi.nlm.nih.gov/pubmed/?term=Pertea+2001+splice) 2001 Mar 1;29(5):1185-90 | Splice site prediction method^#^ |
| *Human Splicing Finder (HSF), version 2.4.1 | Desmet et al. [Human Splicing Finder: an online bioinformatics tool to predict splicing signals](https://www.ncbi.nlm.nih.gov/pubmed/19339519). Nucleic Acid Research (2009) 37 (9): e67. | Splice site prediction method^#^ |
| ^§^Ensembl Compara | Herrero et al. Ensembl comparative genomics resources, *Database*, Volume 2016, 1 January 2016, bav096, <https://doi.org/10.1093/database/bav096> | Evolutionary amino acid conservation |
| UniProt | http://www.uniprot.org/ | Protein domains analysis |
| Amino acid properties | <http://www.russelllab.org/aas/> | Amino acid properties analysis |
| PubMed | https://www.ncbi.nlm.nih.gov/pubmed | Literature search |
| OMIM | <http://omim.org/> | Literature search |
| Mutation/variant databases | <https://www.ncbi.nlm.nih.gov/SNP/>  <http://exac.broadinstitute.org>  https://www.ncbi.nlm.nih.gov/clinvar/  <http://www.lovd.nl/3.0/home>  http://www.umd.be | Literature search  Allele frequency |

*This prediction tool is integrated in Alamut® Visual 2.9.0

^§^By default, orthologues aligned and displayed in Alamut® Visual are taken from the [Ensembl Compara](http://www.ensembl.org/info/genome/compara/index.html) database.

^#^The Splicing Prediction Module of Alamut® Visual 2.9.0 allows setting of user-defined thresholds for displaying splice site prediction scores. A threshold =0 was used as default for all Splice Site prediction programs.

Variants identified in this study have been submitted to the following databases:

LOVD3 Shared Database (ACTA2, ELN, FBN1, FBN2, MYH11, MYLK, NOTCH1, PRKG1, SLC2A10): [https://databases.lovd.nl/shared/genes/](https://databases.lovd.nl/shared/genes/ACTA2)

Loeys-Dietz Syndrome Mutation Database (SMAD2, SMAD3, TGFB2, TGFB3, TGFBR1, TGFBR2): <http://143.169.238.105/LOVD/genes/>

Ehlers-Danlos Syndrome Variant Database (COL3A1): <https://eds.gene.le.ac.uk/home.php?select_db=COL3A1>

Detailed protocols are available upon request.

**References**

Li, H., & Durbin, R. (2010). Fast and accurate long-read alignment with Burrows-Wheeler transform. *Bioinformatics, 26*(5), 589-595. doi:10.1093/bioinformatics/btp698

Li, H., Handsaker, B., Wysoker, A., Fennell, T., Ruan, J., Homer, N., . . . Genome Project Data Processing, S. (2009). The Sequence Alignment/Map format and SAMtools. *Bioinformatics, 25*(16), 2078-2079. doi:10.1093/bioinformatics/btp352

McKenna, A., Hanna, M., Banks, E., Sivachenko, A., Cibulskis, K., Kernytsky, A., . . . DePristo, M. A. (2010). The Genome Analysis Toolkit: a MapReduce framework for analyzing next-generation DNA sequencing data. *Genome Res, 20*(9), 1297-1303. doi:10.1101/gr.107524.110

Richard, S., Aziz, N., Bale, S., Bick, D., Das, S., Gastier-Foster, J., …, [ACMG Laboratory Quality Assurance Committee](https://www.ncbi.nlm.nih.gov/pubmed/?term=ACMG%20Laboratory%20Quality%20Assurance%20Committee%5BCorporate%20Author%5D). (2015). Standards and guidelines for the interpretation of sequence variants: a joint consensus recommendation of the American College of Medical Genetics and Genomics and the Association for Molecular Pathology. [*Genet Med*](https://www.ncbi.nlm.nih.gov/pubmed/25741868) 17(5):405-24
